# Measures of Early-life Behavior and Later Psychopathology in the LifeCycle Project - EU Child Cohort Network: A Cohort Description

**DOI:** 10.2188/jea.JE20210241

**Published:** 2023-06-05

**Authors:** Johanna L. Nader, Mònica López-Vicente, Jordi Julvez, Monica Guxens, Tim Cadman, Ahmed Elhakeem, Marjo-Riitta Järvelin, Nina Rautio, Jouko Miettunen, Hanan El Marroun, Maria Melchior, Barbara Heude, Marie-Aline Charles, Tiffany C. Yang, Rosemary R. C. McEachan, John Wright, Kinga Polanska, Jennie Carson, Ashleigh Lin, Sebastian Rauschert, Rae-Chi Huang, Maja Popovic, Lorenzo Richiardi, Eva Corpeleijn, Marloes Cardol, Tuija M. Mikkola, Johan G. Eriksson, Theodosia Salika, Hazel Inskip, Johan Lerbech Vinther, Katrine Strandberg-Larsen, Kathrin Gürlich, Veit Grote, Berthold Koletzko, Marina Vafeiadi, Jordi Sunyer, Vincent W. V. Jaddoe, Jennifer R. Harris

**Affiliations:** 1 Department of Genetics and Bioinformatics, Division of Health Data and Digitalisation, Norwegian Institute of Public Health, Oslo, Norway; 2 ISGlobal, Instituto de Salud Global de Barcelona, Barcelona, Spain; 3 Department of Child and Adolescent Psychiatry, Erasmus University Medical Center, Rotterdam, The Netherlands; 4 Institut d’Investigació Sanitària Pere Virgili, Hospital Universitari Sant Joan de Reus, Reus, Spain; 5 MRC Integrative Epidemiology Unit at University of Bristol, Population Health Sciences, Bristol Medical School, Bristol, United Kingdom; 6 Center for Life Course Health Research, University of Oulu, Oulu, Finland; 7 Medical Research Center Oulu, Oulu University Hospital and University of Oulu, Oulu, Finland; 8 Department of Pediatrics, Erasmus University Medical Center, Rotterdam, The Netherlands; 9 The Generation R Study Group, Erasmus University Medical Center, Rotterdam, The Netherlands; 10 Institut national de la santé et de la recherche médicale, Institut Pierre Louis d’Épidémiologie et de Santé Publique, Sorbonne Université, Paris, France; 11 Centre for Research in Epidemiology and Statistics, Institut national de la santé et de la recherche médicale, Institut national de la recherche agronomique, Université de Paris, Paris, France; 12 Unité mixte Inserm-Ined-EFS Elfe, Institut national d’études démographiques, Paris, France; 13 Bradford Institute for Health Research, Bradford Teaching Hospitals NHS Foundation Trust, Bradford, United Kingdom; 14 Department of Hygiene and Epidemiology, Medical University of Lodz, Lodz, Poland; 15 Telethon Kids Institute, University of Western Australia, Perth, Australia; 16 Cancer Epidemiology Unit, Department of Medical Sciences, University of Turin and CPO Piemonte, Turin, Italy; 17 Department of Epidemiology, University Medical Center Groningen, Groningen, The Netherlands; 18 Folkhälsan Research Center, Helsinki, Finland; 19 Clinicum, Faculty of Medicine, University of Helsinki, Uusimaa, Finland; 20 Public Health Promotion Unit, National Institute for Health and Welfare, Helsinki, Finland; 21 Department of General Practice and Primary Health Care, University of Helsinki and Helsinki University Hospital, Helsinki, Finland; 22 Singapore Institute for Clinical Sciences, Agency for Science, Technology, and Research, Singapore; 23 Department of Obstetrics & Gynaecology, Yong Loo Lin School of Medicine, National University of Singapore, Singapore; 24 Medical Research Council Lifecourse Epidemiology Unit, Southampton General Hospital, University of Southampton, Southampton, United Kingdom; 25 NIHR Southampton Biomedical Research Centre, University Hospital Southampton NHS Foundation Trust, University of Southampton, Southampton, United Kingdom; 26 Section of Epidemiology, Department of Public Health, University of Copenhagen, Copenhagen, Denmark; 27 Division of Metabolic and Nutritional Medicine, Department of Pediatrics, Dr von Hauner Children’s Hospital, University Hospital, Ludwig Maximilian University of Munich, Munich, Germany; 28 Department of Social Medicine, Faculty of Medicine, University of Crete, Heraklion, Greece; 29 Division of Health Data and Digitalization, Center for Fertility and Health and Department of Genetics and Bioinformatics, The Norwegian Institute of Public Health, Oslo, Norway

**Keywords:** birth and pregnancy cohorts, child behavior and mental health, population epidemiology, child development, DataSHIELD

## Abstract

**Background:**

The EU LifeCycle Project was launched in 2017 to combine, harmonize, and analyze data from more than 250,000 participants across Europe and Australia, involving cohorts participating in the EU-funded LifeCycle Project. The purpose of this cohort description is to provide a detailed overview of the major measures within mental health domains that are available in 17 European and Australian cohorts participating in the LifeCycle Project.

**Methods:**

Data on cognitive, behavioral, and psychological development has been collected on participants from birth until adulthood through questionnaire and medical data. We developed an inventory of the available data by mapping individual instruments, domain types, and age groups, providing the basis for statistical harmonization across mental health measures.

**Results:**

The mental health data in LifeCycle contain longitudinal and cross-sectional data from birth throughout the life course, covering domains across a wide range of behavioral and psychopathology indicators and outcomes, including executive function, depression, ADHD, and cognition. These data span a unique combination of qualitative data collected through behavioral/cognitive/mental health questionnaires and examination, as well as data from biological samples and indices in the form of imaging (MRI, fetal ultrasound) and DNA methylation data. Harmonized variables on a subset of mental health domains have been developed, providing statistical equivalence of measures required for longitudinal meta-analyses across instruments and cohorts.

**Conclusion:**

Mental health data harmonized through the LifeCycle project can be used to study life-course trajectories and exposure-outcome models that examine early life risk factors for mental illness and develop predictive markers for later-life disease.

## BACKGROUND AND PURPOSE

Effects of early-life exposures on later-life mental health are well known, but more research to understand and elucidate the pathways from stressors to outcomes is needed. The LifeCycle Project - EU Child Cohort Network, a Horizon 2020 project, is a pan-European and Australian initiative comprised of 19 pregnancy and birth cohorts, established to study exposure-to-outcome associations and trajectories across the life course (https://lifecycle-project.eu/).^1^ In general, studies in LifeCycle aim to construct developmental trajectories, develop risk assessment models, measure developmental adaptations, and evaluate mediating epigenetic effects to better understand the consequences of early-life exposures to stressors for risk factors and diseases in adulthood. The large sample sizes achieved through this consortium facilitate high statistical power needed for increased accuracy of estimates and more robust findings.

Mental health is one of the main outcomes within the LifeCycle Project.^1^ While mortality rates for many non-communicable diseases have steadily declined in some populations over the past few decades, such as coronary heart disease^2^^,^^3^ and chronic obstructive pulmonary disease,^4^ the global burden of mental illness is on the rise.^5^ The impact of mental illness on disability and socioeconomic prosperity is increasing around the world, and it is predicted that mental illness will contribute more to disability-adjusted life years (DALYs) than any other category of diseases by the year 2030.^6^ An understanding of how mental health impacts and mediates disease risk and prognosis for other conditions is also beginning to emerge, with recent meta-analyses revealing significantly higher risks for cardiovascular^7^ and metabolic^8^ diseases linked to severe mental illness.

This cohort description focuses on the extensive work done to catalogue and harmonize variables related to cognitive, behavioral, and psychological development within the broader LifeCycle consortium.^1^ It is well-recognized that experiences in early life play an important part in shaping later mental health,^9^ and the data within the LifeCycle Project permit analyses of these associations. LifeCycle includes many pregnancy and birth cohorts that prospectively collected data on offspring from conception and across different ages of child, adolescent, and adult development. The availability of data from multiple follow-up assessments is essential for probing questions about causality and linking early-life stressors with later life mental health symptoms and outcomes.

**Figure 1.**
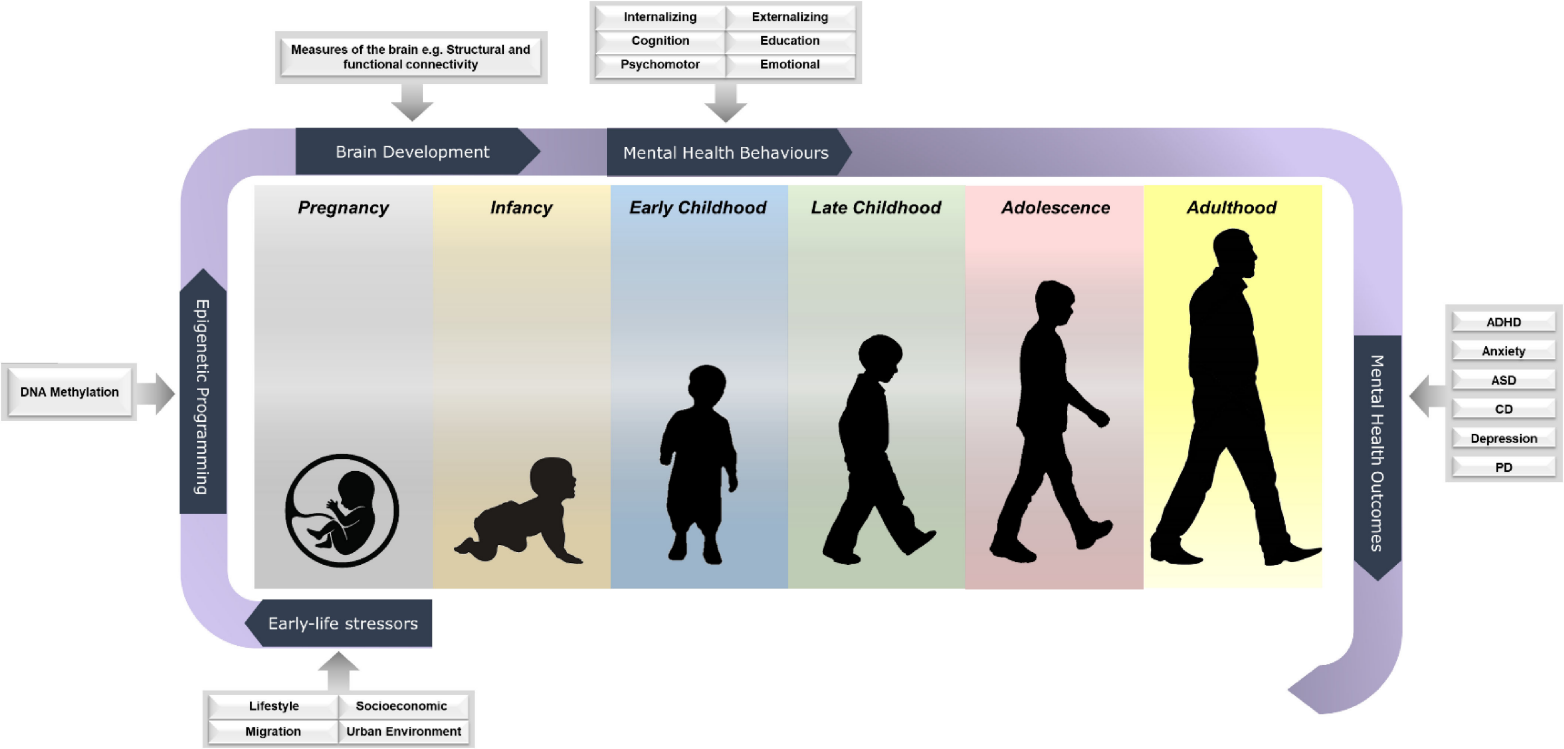
Available mental health outcomes from prenatal to adulthood in the LifeCycle mental health and cognitive data. ADHD, attention deficit hyperactivity disorder; ASD, autism spectrum disorders; CD, cognitive disorders; PD, psychiatric disorders.

The mental health studies in LifeCycle aim to investigate epidemiological interrelations between early-life exposures, behavior, and cognition, with later mental and physical health. Towards this end we have harmonized measures from 17 LifeCycle cohorts to enable studies that examine how environmental stressors in utero and in early childhood affect, or are associated with, psychological trajectories, behaviors, and mental outcomes throughout childhood, adolescence, and adulthood. Additionally, we are examining the nature and degree of mediation of these associations through epigenetic changes and brain development (Figure 1). To our knowledge, the data compiled for these studies within LifeCycle represents the largest ongoing consolidation of childhood behavior, psychopathology, and cognition data to date, encompassing more than 200 multidimensional and multi-informant established mental health measures collected from at least 250,000 participants.

## METHODS

### Cohorts, participants, and follow-up

A total of 17 child-parent cohorts based in 13 countries are contributing mental health data: Avon Longitudinal Study of Parents and Children (ALSPAC; United Kingdom), Born in Bradford (BiB; United Kingdom), EU Childhood Obesity Programme (CHOP; Germany/Italy/Spain/Poland/Belgium), Danish National Birth Cohort (DNBC; Denmark), Etude des Déterminants du développement et de la santé de l’Enfant (EDEN; France), Etude Longitudinale Française depuis l’Enfance (ELFE; France), Groningen Expert Center for Kids with Obesity Drenthe cohort (GECKO Drenthe cohort; The Netherlands), the Generation R Study (Generation R; The Netherlands), Helsinki Birth Cohort Study (HBCS; Finland), Infancia y Medio Ambiente (INMA; Spain), The Norwegian Mother, Father and Child Cohort Study (MoBa; Norway), Northern Finland Birth Cohorts (NFBC1966/1986; Finland), Nascita e INFanzia: gli Effetti dell’Ambiente (NINFEA; Italy), The Raine Study (Australia), Rhea Mother & Child Cohort Study (RHEA; Greece), and the Southampton Women’s Survey (SWS; United Kingdom).

**Figure 2.**
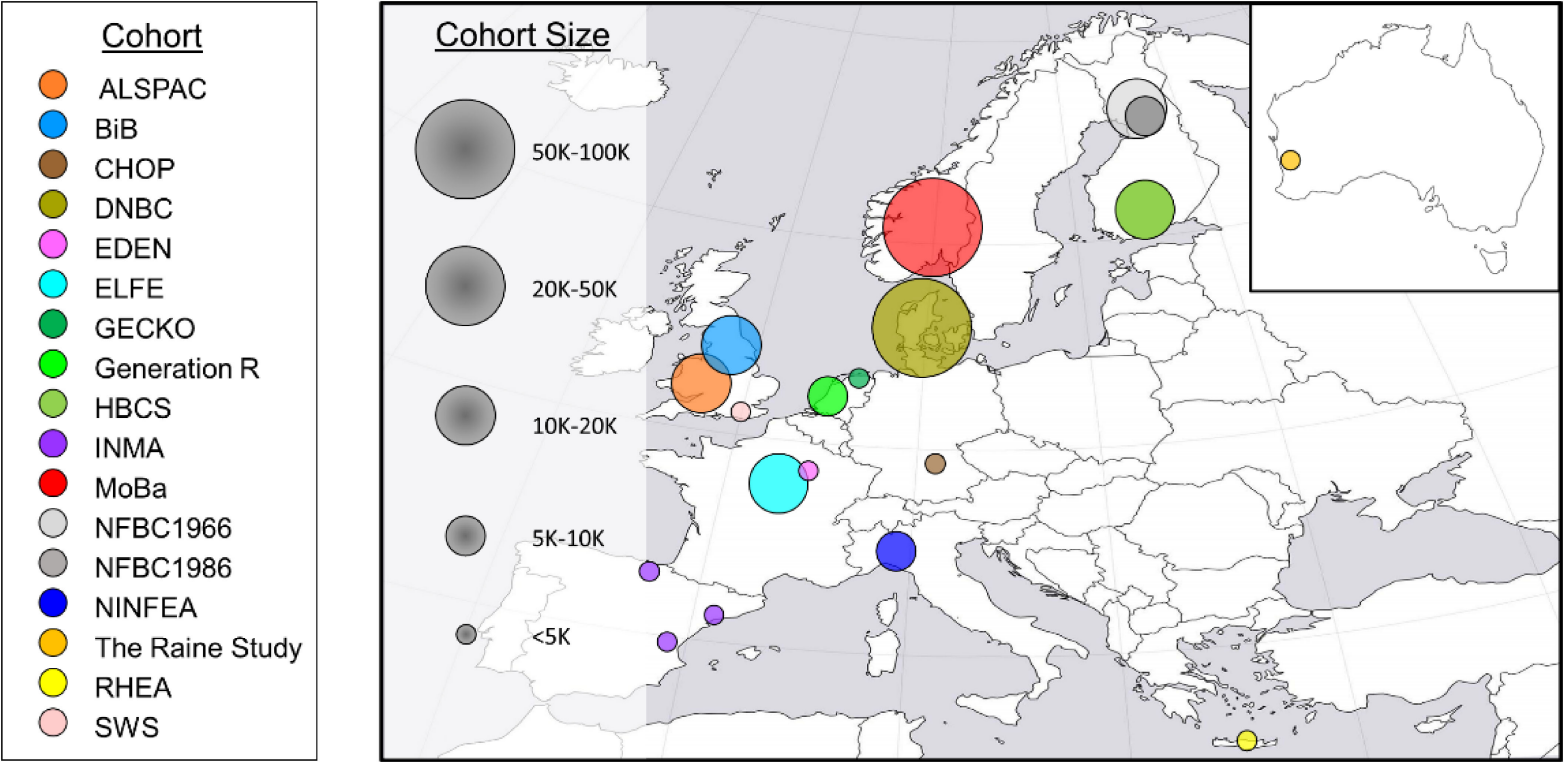
Geographic distribution and sample sizes of cohorts in LifeCycle contributing mental health data. ALSPAC, Avon Longitudinal Study of Parents and Children; BiB, Born in Bradford; CHOP, EU Childhood Obesity Programme; DNBC, Danish National Birth Cohort; EDEN, Etude des Déterminants du développement et de la santé de l’Enfant; ELFE, Etude Longitudinale Française depuis l’Enfance; GECKO, Groningen Expert Center for Kids with Obesity Drenthe cohort; Generation R, the Generation R Study; HBCS, Helsinki Birth Cohort Study; INMA, Infancia y Medio Ambiente; MoBa, The Norwegian Mother, Father and Child Cohort Study; NFBC1966/1986, Northern Finland Birth Cohorts; NINFEA, Nascita e INFanzia: gli Effetti dell’Ambiente; RHEA, Rhea Mother & Child Cohort Study; SWS, and the Southampton Women’s Survey.

The geographic coverage is broad, spanning across much of northern, western, central, and southern Europe, as well as Western Australia (Figure 2). Mental health data from more than 250,000 children are available (as of June 2021), including either mother-child or mother-father-child cohorts, and the study population is diverse with respect to the age of the participants, cohort types, and data collection periods (Table 1). As described elsewhere for the LifeCycle consortium, most of the cohorts in the LifeCycle project (ALSPAC, CHOP, DNBC, EDEN, GECKO, HBCS, INMA, MoBa, NFBC1966/1986, NINFEEA, RHEA, and SWS) predominantly represent ethnic groups from the background population (more than 95% European/White), but certain cohorts like BiB, ELFE, The Generation R Study, and The Raine Study have significant representation of other ethnic groups as well.^10^

**Table 1.** Summary characteristics of LifeCycle cohorts participating with mental health data

Cohort	Location of Coordinating Centre	Cohort Type	Data collection period	Recruitment	*N* (Live Births)
ALSPAC^11^^,^^12^	Avon, United Kingdom	Population-based	1990–present	Pregnancy	14,953
BiB^13^	Bradford, United Kingdom	Population-based	2007–2010	Pregnancy	13,786
CHOP^14^	Belgium (Liege, Brussels), Germany (Munich, Nuremberg), Italy (Milano), Poland (Warsaw), Spain (Reus, Tarragona)	Mixed (Randomised controlled intervention trial (first year) with birth cohort)	2002–2015	First 8 weeks of life	1,678
DNBC^15^	Copenhagen, Denmark	Population-based	1996–present	Pregnancy	96,804
EDEN^16^	Nancy and Poitiers, France	Population-based	2003–2017	Pregnancy	1,907
ELFE^17^	Paris, France	Population-based	2011–present	Birth	18,329
GECKO^18^	Drenthe, The Netherlands	Population-based	2006–present	Pregnancy	2,844
The Generation R Study^19^^,^^20^	Rotterdam, The Netherlands	Population-based	2002–present	Pregnancy	9,749
HBCS^21^	Helsinki, Finland	Population-based	1934–present	Birth	13,345
INMA^22^	Sabadell, Spain	Population-based	2004–present	Pregnancy	622
	Valencia, Spain	Population-based	2003–present	Pregnancy	787
	Gipuzkoa, Spain	Population-based	2006–present	Pregnancy	612
MOBA^23^	Oslo, Norway	Population-based	1999–present	Pregnancy	113,564
NFBC1966^24^	Oulu, Finland	Population-based	1966–present	Pregnancy	12,058
NFBC1986^25^	Oulu, Finland	Population-based	1985/1986–present	Pregnancy	9,432
NINFEA^26^	Torino, Italy	Population-based (Internet- based recruitment)	2005–present	Pregnancy (Internet- based recruitment)	6,816
The Raine Study^27^	Perth, Australia	Population-based (Randomised assignment to multiple ultrasounds during pregnancy)	1989–present	Pregnancy	2,868
RHEA^28^	Crete, Greece	Population-based	2007–present	Pregnancy	1,458
SWS^29^	Southampton, United Kingdom	Population-based	1998–present	Pre-pregnancy	3,158

The participating cohorts include child participants with follow-up data ranging from birth until adulthood (Table 2). Questionnaires, medical records, doctor diagnoses, and registries were variably used across the cohorts to collect data at different ages, but all of the cohorts collected baseline data during pregnancy or at birth and included a follow-up data collection at least once by the time the child participant was 24 months of age. Although the regularity of follow-up differs substantially across cohorts, ranging from annually to many years apart, at least half of the cohorts performed some type of follow-up data collection for all incremental age groups up until 6 years of age. The overlapping age ranges enable comprehensive comparative analyses of mental health constructs between and within the populations to which these index children belong.

**Table 2.** Age ranges and sex (% male:female) of participants during assessment in LifeCycle cohorts

Cohort	Baseline (no. live births)	Age of child at assessment (years)														

		0 to <1	1 to <2	2 to <3	3 to <4	4 to <5	5 to <6	6 to <7	7 to <8	8 to <9	9 to <10	10 to <12	12 to <14	14 to <16	16 to <18	18+
ALSPAC^a^	14,953	11,466	11,097	9,993	9,779	9,632	8,683	8,410	8,282	7,481	7,718	7,552	6,829	5,506	5,212	
*Sex (% M:F)*		51.6:48.4	51.7:48.3	51.8:48.2	51.7:48.3	51.8:48.2	51.6:48.4	51.4:48.6	50.7:49.3	49.8:50.2	49.3:50.7	49.4:50.6	49.1:50.9	47.1:52.0	43.6:56.4	

BiB	13,786	1,436	3,484	2,911	1,167	2,505	79									
*Sex (% M:F)*	51.6:48.4	49.6:50.4	50.3:49.7	50.1:49.9	47.9:52.1	49.9:50.1	51.9:48.1									

CHOP^b^	1,678	1,175	1,067	934	747	674	655	1,028	594	589		719				
*Sex (% M:F)*	50.7:49.3	49.0:51.0	48.1:51.9	48.2:51.8	46.6:53.4	47.2:52.8	47.2:52.8	48.5:51.5	49.0:51.0	47.0:53.0		46.5:53.5				

DNBC	96,804	70,276	65,548				1,628^c^		57,156			46,345^d^				35,558^f^
*Sex (% M:F)*	51.3:48.7	51.1:48.9	51.0:49.0				52.0:48.0		51.2:48.8			49.7:50.3				41.6:58.4
												48,579^e^				
												48.2:51.8				

EDEN^g^	1,907		1,612	1,429	1,257	1,192	1,114					557				
*Sex (% M:F)*			52.8:47.2	52.2:47.8	52.4:47.6	51.3:48.7	52.7:47.3					51.3:48.7				

ELFE	18,329	16,547	14,439	13,277	11,935											
*Sex (% M:F)*	51.4:48.6	51.2:48.9	51.2:48.8	50.7:49.3	51.2:48.8											

GECKO	2,844	2,812	2,558	2,319	1,819	1,486	2,322					2,299				
*Sex (% M:F)*		50.3:49.7	50.3:49.7	50.1:49.9	51.2:48.8	51.4:48.6	50.3:49.7					49.8:50.2				

Generation R	9,749	7,893					8,305				7,393		6,842			
*Sex (% M:F)*	50.7:49.3	50.5:49.5					50.5:49.5				50.1:49.9		50.3:49.7			

HBCS	13,345	13,345	13,342	13,342	8,947	7,252	9,947	10,055	10,046	10,033	9,985	9,902				13,345
*Sex (% M:F)*	52.3:47.7	52.3:47.7	52.3:47.7	52.3:47.7	52.0:48.0	51.7:48.3	52.6:47.4	52.7:47.3	52.6:47.4	52.7:47.3	52.8:47.2	52.8:47.2				52.3:47.7

INMA-Sabadell	622		559			481		473			433					
*Sex (% M:F)*			51.3:48.7			51.4:48.7		51.6:48.4			52.0:48.0					

INMA-Valencia	787		694				530		469		429					
*Sex (% M:F)*			52.6:47.4				51.7:48.3		50.8:49.3		50.6:49.4					

INMA-Gipuzkoa	612		556	506		394			397	382						
*Sex (% M:F)*	50.3:49.8		49.1:50.9	52.0:48.0		49.2:50.8			49.4:50.6	54.0:46.0						

MoBa	113,564	87,801	74,750		58,835		41,617		53,517	43,609						
*Sex (% M:F)*		51.0:49.0	51.0:49.0		51.0:49.0		50.9:49.1		51.3:48.7	50.9:49.1						

NFBC1966	12,058		10,729											10,927		9,517
*Sex (% M:F)*			50.8:49.2											50.4:49.6		51.3:48.7

NFBC1986	9,432		1,803						8,416^d^					6,985^d^		Data collection ongoing (2019–2020)
*Sex (% M:F)*			50.9:49.1						51.3:48.7					50.0:50.0		
									8,525^h^					7,344^e^		
									51.5:48.5					48.5:51.5		
														6,795^i^		
														49.4:50.6		

NINFEA	7,527^j^	6,907	6,279			4,398			2,348			837				
*Sex (% M:F)*		50.7:49.3				51.1:48.9			50.3:49.7			50.8:49.2				

The Raine Study	2,868		2,430	1,974	2,260		2,236			2,140		2,048		1,864	1,693	1,462
*Sex (% M:F)*	50.7:49.3		50.9:49.1	52.1:47.9	50.9:49.1		51.6:48.4			51.4:48.6		51.7:48.3		51.4:48.6	49.9:50.1	48.9:51.1

RHEA	1,458	1,257	569			904		626								
*Sex (% M:F)*	50.1:49.9	50.2:49.8	54.5:45.5			52.3:47.7		55.1:44.9								

SWS	3,158	2,959	2,875	2,779	2,625	1,182		2,034		1,214						
*Sex (% M:F)*		51.7:48.3	51.9:48.1	51.8:48.2	52.1:47.9	51.9:48.1		51.3:48.7		49.4:50.6						

^a^ALSPAC follow-up data is based on number of parents completing at least some of the questionnaire(s) on young person up to age 7 years, and number of children attending clinic from age 7 years and onwards.

^b^CHOP follow-up data is based on number of children with at least one anthropometric measurement at the considered age.

^c^DNBC follow-up data at 5 years based on a subsample, selected based on parental alcohol characteristics.

^d^Parent-reported data.

^e^Self-reported data.

^f^DNBC data collection for 18-year follow-up is currently ongoing.

^g^EDEN follow-up data is based on number of children with at least one neurodevelopment assessment at the considered age.

^h^Teacher-reported data.

^i^Clinical data.

^j^NINFEA baseline data refers to no. pregnant women recruited.

### Main outcome measures

#### Psychological, motor, and cognitive measures

**Figure 3.**
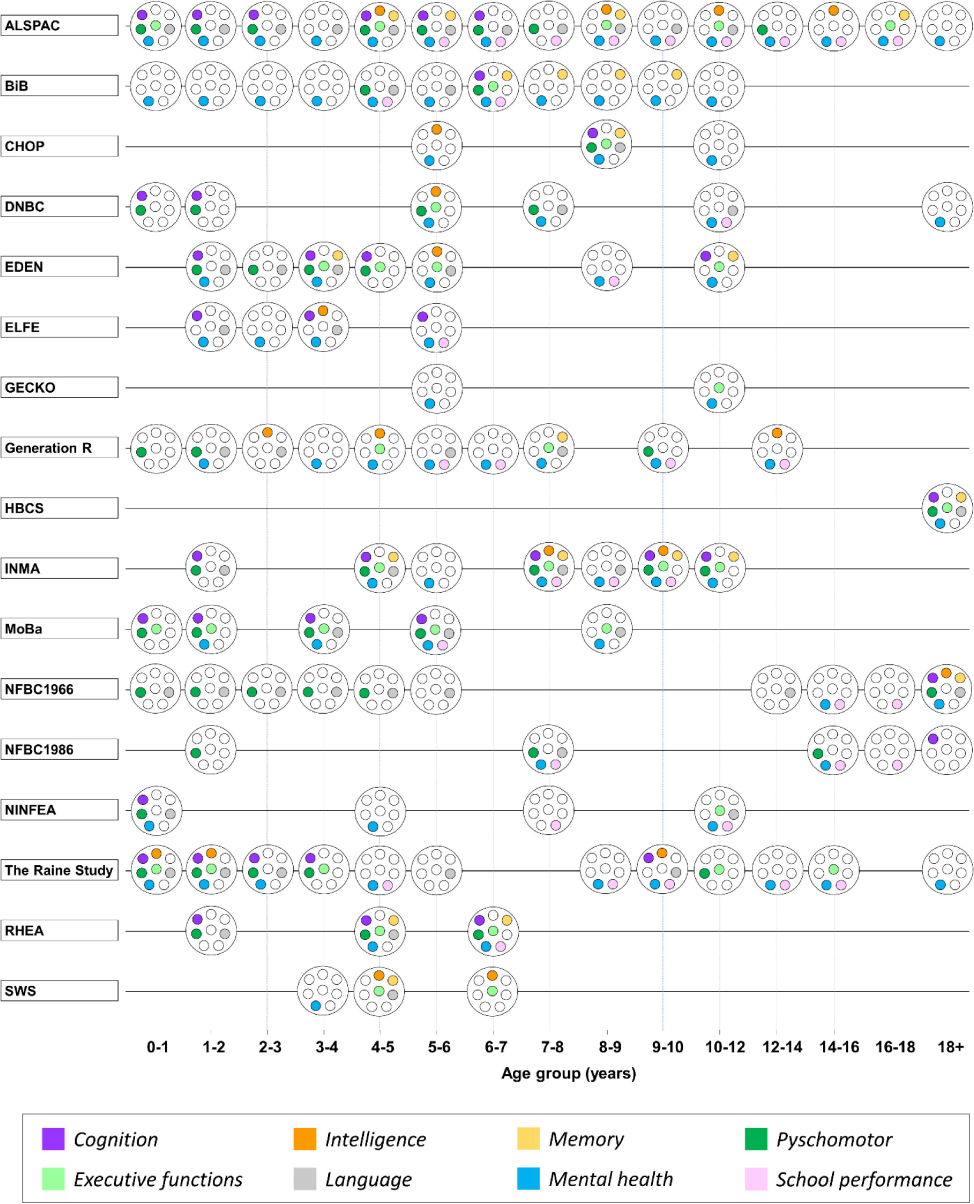
Overview of overlap in LifeCycle mental health, behavioral, and cognitive domains across age

Mental and cognitive disorders comprise some of the most frequently diagnosed conditions in children under 18 years of age. The combined data resource will contain information pertaining to the children from more than 200 mental health measures, covering eight clinical domains across 60 dimensions (eTable 1). A majority of these measures assess domains under a broad banner of ‘mental health’, encompassing psychological, cognitive and behavioral functions and development (67.0%; 136 of 203) and covering dimensions such as neurodevelopmental disorders, internalizing and externalizing symptoms, temperament, and mental diagnoses. Further domains include language skills (31.0%; 63 of 203), executive functions (29.1%; 59 of 203), memory (11.3%; 23 of 203) and general intelligence (8.4%; 17 of 203) (eTable 1). There are many commonalities between mental health domain-types and significant overlap in the age groups with measures in specific domains (Figure 3). This makes it possible to harmonize the data.^30^ Most of the cohorts continue to follow their participants, and the availability of harmonized data will tend to increase with time.

**Figure 4.**
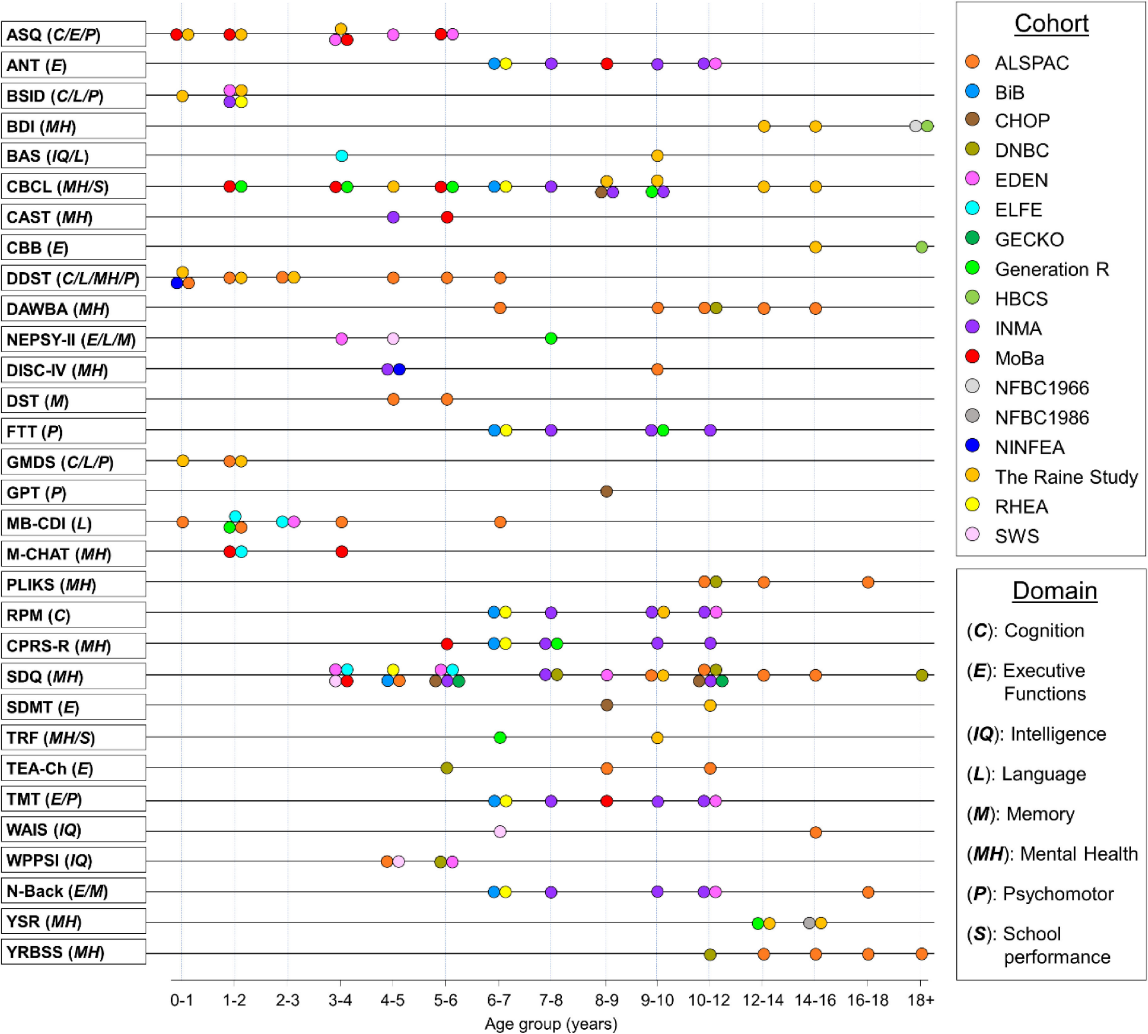
Overview of overlap in mental health and cognitive measures in the LifeCycle cohorts providing mental health data. Summary of overlapping measures and age ranges in participating cohorts. The full list of available measures (including non-overlapping) are described in eTable 1. ANT, Attention Network Task; ASQ, Ages and Stages Questionnaire; BAS, Behavioral Approach System; BDI, Becks Depression Inventory; BRIEF, Behavior Rating Inventory of Executive Function; BSID, Bayley Scales of Infant Development; CAST, Childhood Asperger Syndrome Test; CBB, CogState Brief Battery; CBCL, Child Behavior Checklist; CPRS-R, Revised Conners’ Parent Rating Scale; DAWBA, Development and Well-Being Assessment; DDST, Denver Developmental Screening Test; DISC-IV, Diagnostic Interview Schedule for Children; DST, Digit Span Test; FTT, Finger Tapping Test; GMDS, Griffiths Mental Development scales; GPT, Grooved Pegboard Test; M-CHAT, Modified Checklist for Autism in Toddlers; MB-CDI, MacArthur-Bates Communicative Development Inventories; N-Back, Working Memory Test; NEPSY-II, Developmental NEuroPSYchological Assessment, Second Edition; PLIKS, Psychosis-like symptoms measure; RPM, Raven’s Progressive Matrices; SDMT, Symbol Digit Modalities Test; SDQ, Strengths and Difficulties Questionnaire; TEA-ch, Test of Everyday Attention for Children; TMT, Trail Making Test; TRF, Teacher Report Form; WASI, Wechsler Abbreviated Scale of Intelligence; WPPSI, Wechsler Preschool and Primary Scale of Intelligence; YRBSS, Youth Risk Behavior Surveillance System; YSR, Youth Self-Report.

There are a number of approaches to harmonize data, and several of these have been described and successfully implemented in large collaborations.^10^^,^^31^^–^^33^ The LifeCycle Project has developed a protocol to generate harmonized variables across a selection of important cognitive and mental health domains. This harmonization approach creates standardized scores and percentiles for important domains, such as internalizing and externalizing symptoms, ADHD and ASD symptoms and diagnosis, and language and motor functions. Percentiles and standardized scores were used, as they allow the pooling of mental health outcome data collected using different scales or instruments. One of the biggest harmonization challenges this project faced was obtaining a thorough inventory of the available mental health data in individual cohorts, which was overcome by mapping the available data by instrument, measure, age group, and domain. A subset of cohorts has also employed items from the same mental health, cognitive, and motor function measures, and these data can be pooled or co-analyzed without the need for harmonization (Figure 4). All of the measures harmonized thus far by age and cohort can be found in the LifeCycle online catalogue (https://catalogue.lifecycle-project.eu/).

## PLANNED ANALYSES

### Early-life exposures – lifestyle, migration, socioeconomic, and urban environment

The LifeCycle online catalogue^10^ also contains information on harmonized data on diverse measures of exposures early in life. These will enable the analysis of risk models for mental health that assess the nature and impact of indirect and direct exposures experienced in early life and comorbidities on adverse mental health symptoms and other health conditions. Comprehensive exposure-outcome analyses will also be used to develop predictive markers for mental health in children and adolescents, which may help shape the prediction of mental disorders, allowing for targeted early intervention.

### Mediating pathways - brain development

Early life is a particularly vulnerable time-window for brain development. The vital stages of neurogenesis, proliferation, and migration occur almost exclusively during fetal development, and experience-dependent brain connectivity (ie, myelination) is largely shaped and completed in early childhood.^34^ Research-based evidence has repeatedly linked brain structure, volume, and connectivity indicators to a number of behavioral and cognitive outcomes.^35^^–^^37^ However, study samples are often limited in size and population diversity, and only few longitudinal studies exist.^38^ A subset of cohorts in LifeCycle have participant data on structural brain imaging (ALSPAC, *n* = 950; Generation R, *n* ≈ 4,000^20^; NFBC1966, *n* = 1,000; NFBC1986, *n* = 600), and will be contributing information on neuroanatomical markers, such as total brain volume, cortical grey matter, white matter volume, ventricular volume, and volumes of subcortical brain structures, including the hippocampus and amygdala. In addition, structural and functional connectivity metrics have been assessed. Data have been collected through neuroimaging techniques, such as fetal ultrasound and magnetic resonance imaging (MRI) in childhood and adulthood. These data enable LifeCycle to describe changes in structural and functional development of the brain from fetal life and infancy and to subsequently associate this brain development in early life with psychopathology outcomes in childhood, adolescence, and adulthood.

### Mediating pathways - epigenetics

**Figure 5.**
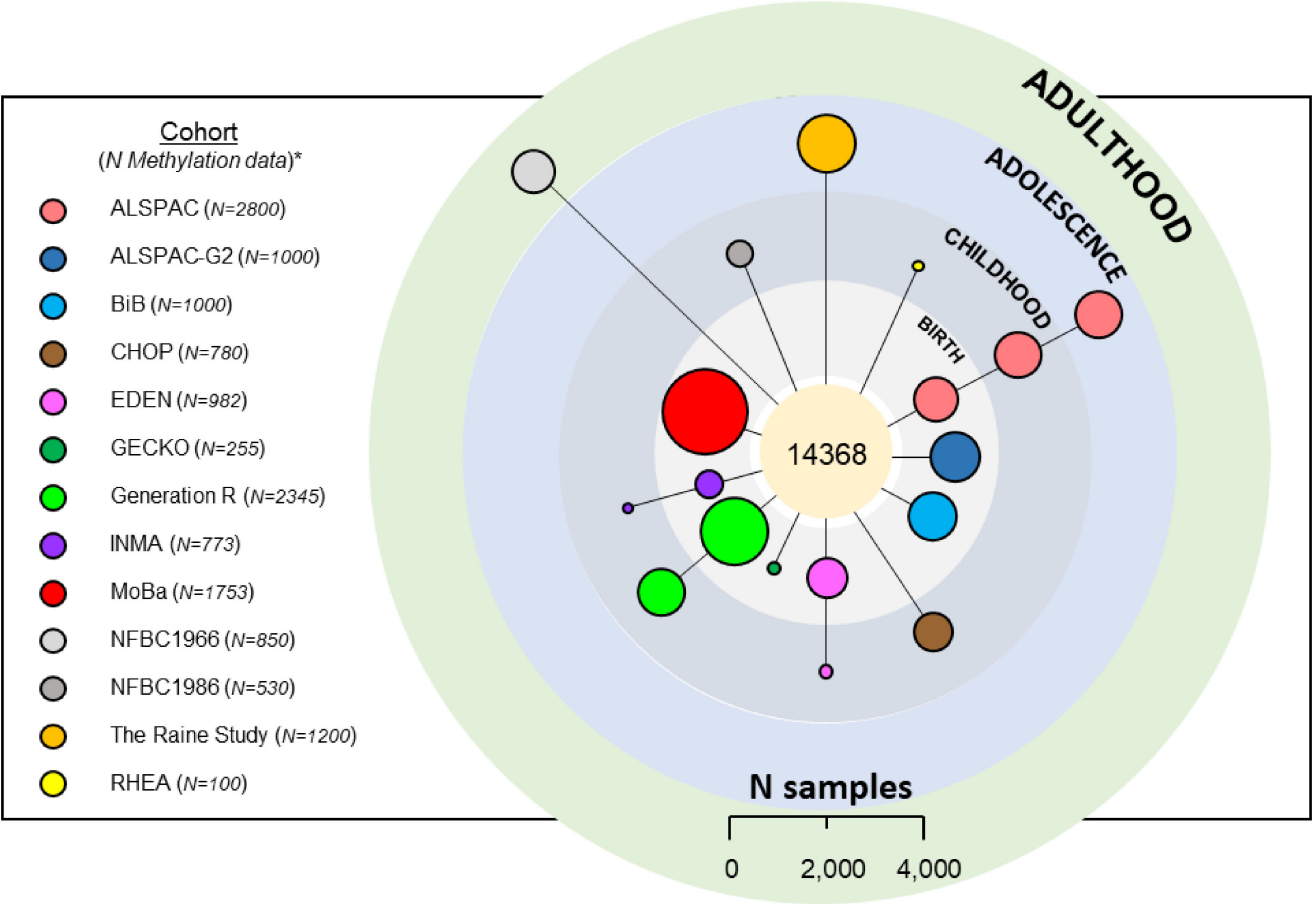
Overview of sample sizes for DNA methylation data in the offspring from birth to adulthood. Circle sizes are proportionate to the DNA methylation sample sizes as indicated in the scale at the bottom of the figure. ^*^Numbers relevant as of June, 2021 (sample processing and data collection is ongoing in several LifeCycle cohorts)

An increasing number of studies are beginning to demonstrate the importance of epigenetic modification in mediating the risk of disease, including mental health outcomes. Epigenetically-modified loci have been linked to a wide range of mental disorders, such as schizophrenia,^39^ as well as childhood onset disorders, such as ADHD^40^ and ASD,^41^ but conflicting and non-replicated associations mean that the causal relationships remain poorly understood.^42^ LifeCycle mental health studies can currently analyze DNA methylation data on 14,368 offspring cohort participants (Figure 5), measured at birth (cord or placenta blood; *N* = 7,783), childhood (0–12 years; *N* = 3,055), adolescence (12–18 years; *N* = 2,680), or adulthood (>18 years; *N* = 850). Six of the thirteen contributing cohorts additionally contain longitudinal epigenetic data (ALSPAC, CHOP [multiple age groups in childhood], EDEN, Generation R, INMA, and RHEA). The particular focus will be to identify epigenetic mechanisms that mediate the effect of early-life exposures on behavioral and cognitive development, as well as mental health outcomes, such as ASD, ADHD, depression, and anxiety. This means it will be possible to track epigenetic changes in participants with behavioral and/or neurodevelopmental outcomes across time and study causal relationships between environmental exposures in pregnancy or early life and later-life mental health outcomes mediated by DNA methylation.

### Framework for collaborative analyses

LifeCycle aims to perform most of the analyses through DataSHIELD.^43^^,^^44^ With the recent launch of the platform and its analytical features for use with LifeCycle harmonized data, a number of novel collaborative studies have begun to form within the theme of mental health. Examples of planned and ongoing exposure-outcome analyses include infant feeding patterns and school-age externalizing behaviors; maternal smoking in pregnancy and adverse child behaviors; associations among sleep, behavior, and cognition; sibling effects and prematurity; and socioeconomic inequalities and general mental health trajectories. Results from these studies are currently pending, but they have already shown that independent participant data resources have been successfully harmonized and can be co-analyzed. The quantity and breadth of mental health and cognitive data available that have been mapped and harmonized by the LifeCycle mental health research group is a singular resource to enable developmental studies of mental health. These data will play an important role in replicating previous findings with enhanced statistical power, expanding upon previous associations through larger and more diverse samples, and in the development of novel models to describe how multi-faceted early-life exposures can shape and influence the landscape of mental health in later life.

## STRENGTHS AND LIMITATIONS

There are many strengths inherent in large consortia such as LifeCycle.^1^ Key among these is that LifeCycle is building the EU Child Cohort Network, a sustainable research network that will enable continued exploitation of the LifeCycle data, metadata, and collaborative progress beyond the usual timelines of a funded grant. Another important strength is the ability to study age differences and age-related mental health and cognitive changes; this developmental aspect will help to understand the long- and short-term consequences of early-life exposures, and how other factors, such as epigenetic changes, may mediate later health outcomes. Geographic diversity is also a key feature; it provides enhanced location coverage and generalizability of results and also facilitates intra- and inter-population comparisons. This makes it possible to make more reliable causal inferences due to different confounding structures.

The number of critical mental health domains covered is another strength, allowing for exposure-outcome research into many important and well-studied areas within this field. The availability of the harmonization protocols, coupled with the extensive overview of mental health measures, including detailed information on the dimensions and age ranges across cohorts, provides users with an integrated catalogue of psychological, cognitive, and psychomotor data in participating cohorts. Furthermore, the use of DataSHIELD enables a flexible and data-secure approach that allows new cohorts and centers to link into the analysis network and contribute with their own data, as well as the addition of newly harmonized data as these are collected and updated. This open-source analysis platform “takes the analysis to the data, not the data to the analysis”, providing researchers with the ability to remotely analyze data from multiple datasets without being able to access the data itself.^44^^,^^45^ Removing the need to physically share data externally means participating cohorts bypass ethical concerns related to the protection of privacy and other issues that arise when participant data are being sent internationally to multiple users, so it addresses some important ethico-legal considerations that are often associated with individual-level data sharing and analysis.

The heterogeneity of the psychological and cognitive measures available presents a potential limitation. Depending on the specific research question under investigation and measurement equivalence of constructs between different instruments, robust harmonisation^30^^,^^32^ of certain measures may not be possible or may be limited to a small number of cohorts. This reduces the sample size or the range of participant ages that are possible to include. Within-country geographical bias of many of the cohorts may also present a weakness. Specifically, the urban-centric nature of many of the studies could mean that the generalizability of findings will be somewhat skewed, and the population-level inferences will need to take this bias into account. Furthermore, DNA methylation and brain imaging data are only available for less than 10% of the total study participants. These smaller sample sizes may limit the number and strength of associations that can be found, as well as the distribution of participant ages and geographic and ethnic origins. However, the cohort studies are continuously expanding and adding new data on their participants, including phenotypic, genetic, epigenetic, and biological data. The collaborative groundwork laid by LifeCycle will make it possible to continue building upon the analyses that have been performed and help to mitigate some of the limitations that have been described.

## DATA ACCESS

LifeCycle has developed an application procedure for data use proposals as described by Jaddoe et al.^1^ It should be noted that approvals for data use and associated fees remain under the purview of the participating cohorts. This is the case regardless of whether one applies through LifeCycle or directly to the cohort, and these practices may vary across cohorts. The project strives to conduct as many analyses as possible within DataSHIELD. DataSHIELD is freely available to download and use (http://www.datashield.ac.uk/). This enables external cohorts to collaborate with LifeCycle and perform co-analyses. For more information, please visit the official website for the LifeCycle Project (https://lifecycle-project.eu/), or refer to the consortium design paper.^1^

In some cases, data sharing and transfer agreements will need to be developed. These may vary due to country-specific practices and restrictions, as outlined by local General Data Protection Regulation (GDPR) legislation. Application procedures directly to cohorts for data can be found at the following websites:

**ALSPAC**
http://www.bristol.ac.uk/alspac/researchers/access/
For more information on the ALSPAC cohort (including data dictionary, ethical considerations, and funding), refer to eMaterials 2.
**BiB**
https://borninbradford.nhs.uk/research/how-to-access-data/
**CHOP**
https://www.birthcohorts.net/birthcohorts/birthcohort/?id=137
**DNBC**
https://www.ssi.dk/English/RandD/Research%20areas/Epidemiology/DNBC/For%20researchers.aspx
**EDEN**
http://eden.vjf.inserm.fr/index.php/fr/contact
**ELFE**
https://www.elfe-france.fr/en/the-research/access-to-data-and-questionnaires/
**GECKO**
http://www.birthcohorts.net/birthcohorts/birthcohort/?id=138
**The Generation R Study**
https://www.generationr.nl/researchers/collaboration/
**INMA**
http://www.proyectoinma.org/presentacion-inma/politica-colaboracion/en_politica-colaboracion.html
**HBCS**
https://thl.fi/en/web/thlfi-en/research-and-expertwork/projects-and-programmes/helsinki-birth-cohort-study-hbcs-idefix
**MoBa**
https://www.fhi.no/en/op/data-access-from-health-registries-health-studies-and-biobanks/data-from-moba/research-and-data-access/
**NFBC1966/1986**
https://www.oulu.fi/nfbc/
**NINFEA**
https://www.progettoninfea.it/contact_us
**The Raine Study**
https://www.rainestudy.org.au/
**RHEA**
http://www.rhea.gr/en/research/data-access/
**SWS**
https://www.mrc.soton.ac.uk/sws/

## SUPPLEMENTARY MATERIAL

eTable 1. Inventory of cognitive, behavioural, and psychological measures in the LifeCycle project (across cohorts)

eMaterials 1. Cohort study specific acknowledgements

eMaterials 2. Cohort study specific funding
