# Supplementary material for: Measures of Early-life Behavior and Later Psychopathology in the LifeCycle Project - EU Child Cohort Network: A Cohort Description
Source: J Epidemiol. 2023 Jun 5;33(6):321–31. doi: 10.2188/jea.JE20210241 (PMC10165218; doi:10.2188/jea.JE20210241)
Supplement: Supplementary file 1 [file je-33-321-s001.pdf]

eTable 1. Inventory of cognitive, behavioural, and psychological measures in the LifeCycle project (across cohorts)

Instrument	Abbreviation	Domains	ALSPAC	BiB	CHOP	DNBC	EDEN	ELFE	GECKO	Generation R	HBCS	INMA	MoBa	NFBC1966	NFBC1986	NINFEA	RAINE	RHEA	SWS
Ages & Stages Questionnaire	ASQ	Cognition					x												
Ages & Stages Questionnaire	ASQ	Cognition											x						
Ages & Stages Questionnaire	ASQ	Cognition															x		
Australian Developmental Record for Infants and Young Children		Cognition															x		
Bayley Scales of Infant Development	BSID	Cognition					x												
Bayley Scales of Infant Development	BSID	Cognition										x							
Bayley Scales of Infant Development	BSID	Cognition															x		
Bayley Scales of Infant Development	BSID	Cognition																x	
Child Development Inventory	CDI	Cognition						x											
Children's developmental progress from birth to five years	STYCAR	Cognition															x		
Denver Developmental Screening Test	DDST	Cognition	x																
Denver Developmental Screening Test	DDST	Cognition														x			
Denver Developmental Screening Test	DDST	Cognition															x		
Early Development Instrument	EDI	Cognition											x						
Early Language Milestone Scale	ELM	Cognition															x		
Gesell Developmental Observation-Revised	GDO-R	Cognition															x		
Griffiths Mental Development scales	GMDS	Cognition	x																
Griffiths Mental Development scales	GMDS	Cognition															x		
Hawaii Early Learning Profile	HELP	Cognition															x		
Hooper Visual Organization Test	HVOT	Cognition			x														
Intelligence Quotient	IQ	Cognition												x					
Matrix Reasoning Test	MRT	Cognition												x					

McCarthy Scales of Children's Abilities	MSCA	Cognition										x							
McCarthy Scales of Children's Abilities	MSCA	Cognition																x	
Mini Mental Status Examination	MMSE	Cognition									x								
Question		Cognition				x													
Question		Cognition				x													
Raven's Progressive Matrices	RPM	Cognition		x															
Raven's Progressive Matrices	RPM	Cognition				x													
Raven's Progressive Matrices	RPM	Cognition										x							
Raven's Progressive Matrices	RPM	Cognition															x		
Raven's Progressive Matrices	RPM	Cognition																x	
Woodside Developmental Screening Chart		Cognition															x		
Abstraction, Inhibition, and Working Memory Task	AIM	Executive functions												x					
Ages & Stages Questionnaire	ASQ	Executive functions					x												
Ages & Stages Questionnaire	ASQ	Executive functions											x						
Ages & Stages Questionnaire	ASQ	Executive functions															x		
Attention Network Test	ANT	Executive functions		x															
Attention Network Test	ANT	Executive functions										x							
Attention Network Test	ANT	Executive functions											x						
Attention Network Test	ANT	Executive functions																x	
Behavior Checklist (clinical rating)		executive functions				x													
Behavior Rating Inventory of Executive Function	BRIEF	Executive functions				x													
Behavior Rating Inventory of Executive Function	BRIEF	Executive functions							x										
Behavior Rating Inventory of Executive Function	BRIEF	Executive functions								x									
Cambridge Neuropsychological Test Automated Battery	CANTAB	Executive functions																	x

Children's Colour Trails Test	CCTT	Executive functions			x														
Cogstate Brief Battery	CBB	Executive functions									x								
Cogstate Brief Battery	CBB	Executive functions									x								
Cogstate Brief Battery	CBB	Executive functions															x		
Conners Kiddie Continuous Performance Test	KCPT	Executive functions										x							
Consortium to Establish a Registry for Alzheimer’s Disease Neuropsychological Battery	CERAD-NB	Executive functions									x								
Continuous Performance Test	CPT	Executive functions			x														
Counting Span Test	CST	Executive functions	x																
Cups task		Executive functions										x							
Developmental Neuropsychological Assessment, Second Editio	NEPSY-II	Executive functions					x												
Developmental Neuropsychological Assessment, Second Editio	NEPSY-II	Executive functions								x									
Developmental Neuropsychological Assessment, Second Editio	NEPSY-II	Executive functions																	x
Doctor diagnosis (self-reported)		Executive functions														x			
Habituation Task	NA	Executive functions	x																
Hungry Donkey Task	HDT	Executive functions			x														
Kaufman Assessment Battery for Children	KABC II	Executive functions			x														
McCarthy Scales of Children's Abilities	MSCA	Executive functions										x							
McCarthy Scales of Children's Abilities	MSCA	Executive functions																x	
Montreal Cognitive Assessment	MoCa	Executive functions									x								
Number Knowledge Test	NKT	Executive functions					x												
Pair Cancellation Test		Executive functions			x														
Reversal Digits Subtest		Executive functions			x														
Stop Signal Task	SST	Executive functions	x																

Stroop Interference Score		Executive functions			x														
Symbol Digit Modalities Test	SDMT	Executive functions			x														
Symbol Digit Modalities Test	SDMT	Executive functions															x		
Teddy Bear Cancellation Test	TBCT	Executive functions					x												
Test of Everyday Attention for Children	TEA-Ch	Executive functions	x																
Test of Everyday Attention for Children	TEA-Ch	Executive functions				x													
Trail Making Test	TMT	Executive functions		x															
Trail Making Test	TMT	Executive functions									x								
Trail Making Test	TMT	Executive functions										x							
Trail Making Test	TMT	Executive functions											x						
Trail Making Test	TMT	Executive functions																x	
Visual Perceptual Skills (non-motor) test		Executive function																	x
Wechsler Preschool and Primary Scale of Intelligence	WPPSI	Executive functions	x																
Wechsler Preschool and Primary Scale of Intelligence	WPPSI	Executive functions				x													
Wechsler Preschool and Primary Scale of Intelligence	WPPSI	Executive functions					x												
Wechsler Preschool and Primary Scale of Intelligence	WPPSI	Executive functions																	x
Working Memory Test	N-Back	Executive functions	x																
Working Memory Test	N-Back	Executive functions		x															
Working Memory Test	N-Back	Executive functions										x							
Working Memory Test	N-Back	Executive functions																x	
British Ability Scales	BAS	Intelligence						x											
British Ability Scales	BAS	Intelligence															x		
Cattell Infant Intelligence Scale	CIIS	Intelligence															x		
Culture Fair Intelligence Test	CFIT	Intelligence			x														
Non-Verbal Intelligence Test	SON-R	Intelligence								x									
Parent Report of Children's Abilities	PARCA	Intelligence								x									

Wechsler Abbreviated Scale of Intelligence	WAIS	Intelligence	x																
Wechsler Abbreviated Scale of Intelligence	WAIS	Intelligence				x													
Wechsler Abbreviated Scale of Intelligence	WAIS	Intelligence																	x
Wechsler Intelligence Scale for Children	WISC	Intelligence	x																
Wechsler Intelligence Scale for Children	WISC	Intelligence								x									
Wechsler Intelligence Scale for Children	WISC	Intelligence										x							
Wechsler Intelligence Scale for Children	WISC	Intelligence															x		
Wechsler Memory Scale	WMS-III	Intelligence												x					
Wechsler Preschool and Primary Scale of Intelligence	WPPSI	Intelligence	x																
Wechsler Preschool and Primary Scale of Intelligence	WPPSI	Intelligence				x													
Wechsler Preschool and Primary Scale of Intelligence	WPPSI	Intelligence					x												
Wechsler Preschool and Primary Scale of Intelligence	WPPSI	Intelligence																	x
Auditory Analysis Test		Language	x																
Australian Developmental Record for Infants and Young Children		Language															x		
Battelle Developmental Inventory Screening Test	BDIST	Language										x							
Bayley Scales of Infant Development	BSID	Language					x												
Bayley Scales of Infant Development	BSID	Language										x							
Bayley Scales of Infant Development	BSID	Language															x		
Bayley Scales of Infant Development	BSID	Language																x	
British Ability Scales	BAS	Language						x											
British Ability Scales	BAS	Language															x		
British Picture Vocabulary Scale	BPVS	Language		x															

Bus Story Test	BST	Language	x																
Checklist of 20 Statements about Language-Related Difficulties	Språk20	Language											x						
Children's auditory performance scale	CHAPS	Language	x																
Children's Communication Checklist	CCC	Language	x																
Children's Communication Checklist	CCC-2	Language											x						
Children's developmental progress from birth to five years	STYCAR	Language															x		
Children's Test of Nonword Repetition	CNRep	Language	x																
Clinical Evaluation of Language Fundamentals	CELF	Language															x		
Consortium to Establish a Registry for Alzheimer’s Disease Neuropsychological Battery	CERAD-NB	Language								x									
Dale and Bishop language rating		Language											x						
Denver Developmental Screening Test	DDST	Language	x																
Denver Developmental Screening Test	DDST	Language														x			
Denver Developmental Screening Test	DDST	Language															x		
Developmental Neuropsychological Assessment, Second Editio	NEPSY-II	Language					x												
Developmental Neuropsychological Assessment, Second Editio	NEPSY-II	Language								x									
Developmental Neuropsychological Assessment, Second Editio	NEPSY-II	Language																	x
Doctor diagnosis (self-reported)		Language														x			
Early Language in Victoria Study	ELVS	Language											x						
Early Language Milestone Scale	ELM	Language															x		
Early Years Foundation Stage Profile	EYFSP	Language		x															
European oral language test battery of aphasic children	ELOLA	Language					x												

FAS Animals Verbal Fluency Test	FAS	Language			x														
Gesell Developmental Observation-Revised	GDO-R	Language															x		
Griffiths Mental Development scales	GMDS	Language	x																
Griffiths Mental Development scales	GMDS	Language															x		
Hawaii Early Learning Profile	HELP	Language															x		
Initial Consonants Detection Test		Language	x																
Intelligibility/Complexity of 3-year-old Children’s Utterances		Language											x						
Language skills		Language												x					
Language skills		Language													x				
Letter Identification		Language		x															
Loss of Skills		Language											x						
MacArthur-Bates Communicative Development Inventories	MB-CDI	Language	x																
MacArthur-Bates Communicative Development Inventories	MB-CDI	Language					x												
MacArthur-Bates Communicative Development Inventories	MB-CDI	Language						x											
MacArthur-Bates Communicative Development Inventories	MB-CDI	Language								x									
McCarthy Scales of Children's Abilities	MSCA	Language										x							
McCarthy Scales of Children's Abilities	MSCA	Language																x	
Montreal Cognitive Assessment	MoCa	Language									x								
Multisyllabic Word Repetition		Language	x																
Non-word repetition		Language	x																
Object Naming Assessment		Language	x																
Parent Report of Children's Abilities	PARCA	Language								x									

Peabody Picture Vocabulary Test	PPVT	Language															x		
Phonological production		Language	x																
Phonological production		Language		x															
Question		Language		x															
Question		Language				x													
Question		Language											x						
Question		Language											x						
Question		Language											x						
Reynell Developmental Language Scale	RDLS	Language	x																
Semantic Verbal Fluency Test	SVF	Language										x							
Speech and Language Assessment Scale	SLAS	Language											x						
Taaltest Voor Kinderen	TVK	Language								x									
Verbal Fluency Test	VFT	Language												x					
Wechsler Objective Language Dimensions	WOLD	Language	x																
Wechsler Objective Reading Dimensions	WORD	Language	x																
Woodside Developmental Screening Chart		Language															x		
California Verbal Learning Test	CVLT	Memory												x					
Consortium to Establish a Registry for Alzheimer's Disease Neuropsychological Battery	CERAD-NB	Memory									x								
Corsi		Memory		x															
Counting Span Test	CST	Memory	x																
Developmental Neuropsychological Assessment, Second Editio	NEPSY-II	Memory					x												
Developmental Neuropsychological Assessment, Second Editio	NEPSY-II	Memory								x									

Developmental Neuropsychological Assessment, Second Editio	NEPSY-II	Memory																	x
Digit Span Test	DST	Memory	x																
Digit Span Test	DST	Memory											x						
Forward and backwards digit recall		Memory		x															
McCarthy Scales of Children's Abilities	MSCA	Memory										x							
McCarthy Scales of Children's Abilities	MSCA	Memory																x	
Non-word repetition		Memory	x																
Recall of Objects Test		Memory			x														
Reversal Digits Subtest		Memory			x														
Rey Auditory Verbal Learning Test	RAVLT	Memory			x														
Visual Object Learning Test	VOLT	Memory											x						
Wechsler Memory Scale	WMS-III	Memory											x						
Working Memory Test	N-Back	Memory	x																
Working Memory Test	N-Back	Memory		x															
Working Memory Test	N-Back	Memory										x							
Working Memory Test	N-Back	Memory																x	
14 item measure		Mental health	x																
Adolescent Sexuality Activity Index	ASAI	Mental health	x																
Alarm Distress Baby Scale	ADBB	Mental health					x												
Arnett Inventory of Sensation Seeking	AISS	Mental health	x																
Assessment of Adult – Child Interaction with the Etch-a-Sketch	AMCIES	Mental health	x																
Autism Quotient Questionnaire	AQ	Mental health															x		
Battelle Developmental Inventory Screening Test	BDIST	Mental health										x							
Beck Depression Inventory	BDI	Mental health									x								
Beck Depression Inventory	BDI	Mental health												x					
Beck Depression Inventory	BDI	Mental health															x		
Behavior Rating Inventory of Executive Function	BRIEF	Mental health				x													

Behavior Rating Inventory of Executive Function	BRIEF	Mental health							x										
Behavior Rating Inventory of Executive Function	BRIEF	Mental health								x									
Bipolar II scale	BSDS	Mental health												x					
Body Image Scale	BIS	Mental health															x		
Bradburn Happiness Scale (Affect Balance Scale)	ABS	Mental health															x		
Brief Problem Monitor	BPM	Mental health								x									
Bullying and Friendship Interview Schedule	BFIS	Mental health	x																
California Preschool Social Competence Scale	CPSCS	Mental health										x							
Cambridge Hormones and Moods project Friendship questionnaire		Mental health	x																
Cantril's ladder scale		Mental health				x													
Cantril Self-Anchoring Striving Scale		Mental health				x													
Carey Temperament Scales		Mental health	x																
Center for Epidemiologic Studies Depression Scale	CES-D	Mental health									x								
Child Behaviour Checklist	CBCL	Mental health		x															
Child Behaviour Checklist	CBCL	Mental health			x														
Child Behaviour Checklist	CBCL	Mental health								x									
Child Behaviour Checklist	CBCL	Mental health										x							
Child Behaviour Checklist	CBCL	Mental health											x						
Child Behaviour Checklist	CBCL	Mental health															x		
Child Behaviour Checklist	CBCL	Mental health																x	
Childhood Autism Spectrum Test	CAST	Mental health										x							
Childhood Autism Spectrum Test	CAST	Mental health											x						
Childhood Interview for DSM-IV Borderline Personality Disorder		Mental health	x																
Clinical Interview Schedule - Revised	CISR	Mental health	x																

Cognitive style questionnaire	CSQ	Mental health	x																
Communication and Symbolic Behaviour Scales	CSBS	Mental health											x						
Conflict Tactics Scale		Mental health	x																
Denver Developmental Screening Test	DDST	Mental health	x																
Denver Developmental Screening Test	DDST	Mental health														x			
Denver Developmental Screening Test	DDST	Mental health															x		
Depression Anxiety Stress Scales	DASS	Mental health															x		
Development and Well-Being Assessment	DAWBA	Mental health	x																
Development and Well-Being Assessment	DAWBA	Mental health				x													
Diagnostic Interview Schedule for Children	DISC-IV/DSM	Mental health										x							
Diagnostic Interview Schedule for Children	DISC-IV/DSM	Mental health											x						
Diagnostic Interview Schedule for Children	DISC-IV/DSM	Mental health														x			
Diagnostic Interview Schedule for Children	DISC-IV/DSM	Mental health															x		
Doctor diagnosis (self-reported)		Mental health														x			
Dyadic Adjustment Scale	DAS	Mental health															x		
Dysfunctional Attitude Scale	DAS	Mental health	x																
Early Screening of Autistic Traits Questionnaire	ESAT	Mental health											x						
Eating Disorder Examination	EDE	Mental health															x		
Emotionality, Activity, and Sociability Temperament Survey	EAS	Mental health	x																
Emotionality, Activity, and Sociability Temperament Survey	EAS	Mental health											x						
Eriksen Flanker Task	EFT	Mental health		x								x							
General Anxiety Disorder - 7	GAD-7	Mental health	x																
Generalized Self-Efficacy Scale	GSE	Mental health									x								

Hardy-Gill resilience scale		Mental health									x								
Harter's Self Perception Profile for Children		Mental health	x																
Health records		Mental health		x															
Hypomania Checklist-32	HCL-32	Mental health	x																
Hypomanic Personality Scale	HPS	Mental health												x					
Infant Characteristics Questionnaire - 6 months form	ICQ-6	Mental health											x						
Infant-Toddler Social and Emotional Assessment	ITSEA	Mental health											x						
International Personality Item Pool	IPIP	Mental health	x																
Kessler Psychological Distress Scale	K10	Mental health															x		
Life Orientation Test	LOT-R	Mental health									x								
Life Orientation Test	LOT-R	Mental health												x					
Loss of Skills		Mental health											x						
Major Depression Inventory	MDI	Mental health				x													
McKnight Risk Factor Survey	MRFS	Mental health				x													
McMaster Family Assessment Device	FAD	Mental health															x		
Measure created for ALSPAC		Mental health	x																
Modified Checklist for Autism in Toddlers	M-CHAT	Mental health						x											
Modified Checklist for Autism in Toddlers	M-CHAT	Mental health											x						
National Registry data		Mental health									x								
Neuroticism-Extroversion-Openness Personality Inventory	NEO-PI	Mental health									x								
Non-Verbal Communication Checklist	NVCC	Mental health											x						
Nowicki-Strickland Internal-External scale	NSIE	Mental health	x																
Olweus Bullying Questionnaire		Mental health	x																

Parent/Teacher Rating Scale for Disruptive Behaviour Disorders	RS-DBD	Mental health										x							
Perceived Self-Efficacy Scale (Cowen's)	PSES	Mental health														x			
Perceived Stress Scale	PSS	Mental health														x			
Perceptual Aberration Scale	PAS	Mental health											x						
PLIKS-Q		Mental health				x													
Preschool Play Behaviour Scale	PPBS	Mental health										x							
Psychopathy Screening Device		Mental health	x																
Psychosis-like symptoms measure	PLIKS	Mental health	x																
Psychosis-like symptoms measure	PLIKS	Mental health					x												
PTSD checklist 5	PCL-5	Mental health	x																
Quality of Life Questionnaire	SF36	Mental health								x									
Quality of Life Questionnaire	15-D	Mental health											x						
Question		Mental health										x							
Question		Mental health										x							
Question		Mental health										x							
Question		Mental health										x							
Question		Mental health										x							
Question		Mental health										x							
Question		Mental health										x							
Question		Mental health										x							
Question		Mental health										x							
Question		Mental health										x							
Question		Mental health				x													
Question		Mental health				x													
Question		Mental health				x													
Questionnaire of Eating and Weight Patterns-Parent Report	QEWP-P	Mental health										x							
Resilience scale		Mental health								x									
Revised Conners' Parent Rating Scale	CPRS-R	Mental health		x															
Revised Conners' Parent Rating Scale	CPRS-R	Mental health							x										

Revised Conners' Parent Rating Scale	CPRS-R	Mental health										x							
Revised Conners' Parent Rating Scale	CPRS-R	Mental health											x						
Revised Conners' Parent Rating Scale	CPRS-R	Mental health																x	
Revised Physical Anhedonia Scale	PhAS	Mental health												x					
Revised Rutter Parent Scale for Preschool Children		Mental health	x																
Revised Social Anhedonia Scale	SAS	Mental health												x					
Rutter's Behaviour Scale	RB2	Mental health													x				
Schizoidia Scale		Mental health												x					
Screen for Child Anxiety Related Emotional Disorders	SCARED	Mental health											x						
Self image profile for adolescents	SIP-A	Mental health	x																
Self-perception Profile for Adolescents	SPPA	Mental health															x		
Self-reported antisocial behavior for young children questionnaire		Mental health	x																
Short Mood and Feelings Questionnaire	SMFQ	Mental health	x																
Short Mood and Feelings Questionnaire	SMFQ	Mental health											x						
Short Norwegian Hierarchical Personality Inventory for Children	NHiPIC-30	Mental health											x						
Short OCD Screener	SOCS	Mental health				x													
Social communication disorders checklist	SCDC	Mental health	x																
Social Communication Questionnaire	SCQ	Mental health											x						
Social Responsiveness Scale	SRS	Mental health								x									
Spence Children Anxiety Scale	SCAS	Mental health				x													
Stattin and Kerr Parental Monitoring Measure		Mental health	x																
Strengths and Difficulties Questionnaire	SDQ	Mental health	x																
Strengths and Difficulties Questionnaire	SDQ	Mental health		x															

Strengths and Difficulties Questionnaire	SDQ	Mental health			x														
Strengths and Difficulties Questionnaire	SDQ	Mental health				x													
Strengths and Difficulties Questionnaire	SDQ	Mental health					x												
Strengths and Difficulties Questionnaire	SDQ	Mental health						x											
Strengths and Difficulties Questionnaire	SDQ	Mental health							x										
Strengths and Difficulties Questionnaire	SDQ	Mental health										x							
Strengths and Difficulties Questionnaire	SDQ	Mental health											x						
Strengths and Difficulties Questionnaire	SDQ	Mental health															x		
Strengths and Difficulties Questionnaire	SDQ	Mental health																x	
Strengths and Difficulties Questionnaire	SDQ	Mental health																	x
Strengths and Weaknesses of ADHD symptoms and Normal behavior	SWAN	Mental health													x				
Stress in Children	SiC	Mental health				x													
Structured interview		Mental health	x																
Subtypes of the Antisocial Behavior Questionnaire	STAB	Mental health	x																
Symptom Check List -25	SCL-25	Mental health												x					
Teacher Report Form	TRF	Mental health								x									
Teacher Report Form	TRF	Mental health															x		
Temperament and character inventory	TCI	Mental health												x					
The Children's Eating Behaviour Questionnaire	CEBQ	Mental health											x						
The Emotionality, Activity and Shyness Temperament Questionnaire	EAS	Mental health											x						
The Trier Social Stress Test	TSST	Mental health									x								
Toddler Temperament Questionnaire	TTQ	Mental health															x		
Toronto Alexithymia Scale	TAS	Mental health												x					
Tridimensional Personality Questionnaire	TPQ	Mental health									x								

Tridimensional Personality Questionnaire	TPQ	Mental health												x					
Volume of offending measure used in the Edinburgh Study of Youth Transitions and Crime		Mental health	x																
Warwick-Edinburgh Mental Well-Being Scale	WEMWBS	Mental health				x													
Ways of Coping checklist	WAYS	Mental health												x					
Youth Risk Behavior Surveillance System	YRBSS	Mental health	x																
Youth Risk Behavior Surveillance System	YRBSS	Mental health				x													
Youth Self Report	YSR	Mental health								x									
Youth Self Report	YSR	Mental health													x				
Youth Self Report	YSR	Mental health															x		
Ages & Stages Questionnaire	ASQ	Psychomotor					x												
Ages & Stages Questionnaire	ASQ	Psychomotor											x						
Ages & Stages Questionnaire	ASQ	Psychomotor															x		
Australian Developmental Record for Infants and Young Children		Psychomotor															x		
Backward balancing		Psychomotor								x									
Bayley Scales of Infant Development	BSID	Psychomotor					x												
Bayley Scales of Infant Development	BSID	Psychomotor										x							
Bayley Scales of Infant Development	BSID	Psychomotor															x		
Bayley Scales of Infant Development	BSID	Psychomotor																x	
Brunet-Lezine psychometric scale	BLS	Psychomotor					x												
Children's developmental progress from birth to five years	STYCAR	Psychomotor															x		
Clinical Kinematic Assessment Tool	CKAT	Psychomotor		x															
Denver Developmental Screening Test	DDST	Psychomotor	x																
Denver Developmental Screening Test	DDST	Psychomotor														x			

Denver Developmental Screening Test	DDST	Psychomotor															x		
Developmental Coordination Disorder Questionnaire	DCDQ	Psychomotor				x													
Developmental Coordination Disorder Questionnaire	DCDQ	Psychomotor				x													
Finger Tapping Test	FTT	Psychomotor		x															
Finger Tapping Test	FTT	Psychomotor								x									
Finger Tapping Test	FTT	Psychomotor										x							
Finger Tapping Test	FTT	Psychomotor																x	
Gesell Developmental Observation-Revised	GDO-R	Psychomotor															x		
Griffiths Mental Development scales	GMDS	Psychomotor	x																
Griffiths Mental Development scales	GMDS	Psychomotor															x		
Grooved Pegboard Test	GPT	Psychomotor			x														
Grooved Pegboard Test	GPT	Psychomotor												x					
McCarthy Scales of Children's Abilities	MSCA	Psychomotor										x							
McCarthy Scales of Children's Abilities	MSCA	Psychomotor																x	
Motor skills		Psychomotor	x																
Motor skills		Psychomotor												x					
Motor skills		Psychomotor													x				
Movement Assessment Battery for Children	M-ABC	Psychomotor	x																
Movement Assessment Battery for Children	M-ABC	Psychomotor				x													
Movement Assessment Battery for Children	M-ABC	Psychomotor				x													
Peg Moving Task	PMT	Psychomotor					x												
Purdue Pegboard Test	PPT	Psychomotor															x		
Question		Psychomotor				x													
Question		Psychomotor				x													
Question		Psychomotor											x						

Touwen Infant Neurological Examination	TouwenNE	Psychomotor								x									
Woodside Developmental Screening Chart		Psychomotor															x		
20 items		School performance	x																
Child Behaviour Checklist	CBCL	School performance		x															
Child Behaviour Checklist	CBCL	School performance			x														
Child Behaviour Checklist	CBCL	School performance								x									
Child Behaviour Checklist	CBCL	School performance										x							
Child Behaviour Checklist	CBCL	School performance											x						
Child Behaviour Checklist	CBCL	School performance															x		
Child Behaviour Checklist	CBCL	School performance																x	
CITO Scores		School performance								x									
Early Development Instrument	EDI	School performance											x						
Early Years Foundation Stage Profile	EYFSP	School performance		x															
Grades		School performance												x					
Grades		School performance													x				
Grades		School performance														x			
Key Stage 1 Assessment		School performance		x															
Maths test		School performance	x																
National Pupil Database		School performance	x																
Neale Analysis of Reading Ability	NARA II	School performance	x																
Perceived school performance		School performance				x													
Question		School performance	x																
Question		School performance	x																
Question		School performance				x													
Question		School performance					x												
Question		School performance						x											
Question		School performance											x						
Question		School performance											x						
Question		School performance													x				

Question		School performance													x				
Question		School performance													x				
Rutter's Behaviour Scale	RB2	School performance													x				
Teacher Report Form	TRF	School performance								x									
Teacher Report Form	TRF	School performance															x		
Vineland Adaptive Behaviour Scale-II	VABS-II	School performance											x						

eTable 2. Inventory of cognitive, behavioural, and psychological measures in the LifeCycle project (across age groups)

Measure	Abbreviation	Domains	Cohort	0-1Y	1-2Y	2-3Y	3-4Y	4-5Y	5-6Y	6-7Y	7-8Y	8-9Y	9-10Y	10-12Y	12-14Y	14-16Y	16-18Y	18-20Y	20-25Y	25-30Y	30+ Y
Ages & Stages Questionnaire	ASQ	Cognition	EDEN				x	x													
Ages & Stages Questionnaire	ASQ	Cognition	MoBa	x	x		x		x												
Ages & Stages Questionnaire	ASQ	Cognition	RAINE	x	x		x														
Australian Developmental Record for Infants and Young Children		Cognition	RAINE	x	x																
Bayley Scales of Infant Development	BSID	Cognition	EDEN		x																
Bayley Scales of Infant Development	BSID	Cognition	INMA		x																
Bayley Scales of Infant Development	BSID	Cognition	RAINE	x	x																
Bayley Scales of Infant Development	BSID	Cognition	RHEA		x																
Child Development Inventory	CDI	Cognition	ELFE		x		x		x												
Children's developmental progress from birth to five years	STYCAR	Cognition	RAINE	x	x																
Denver Developmental Screening Test	DDST	Cognition	ALSPAC	x	x	x		x	x	x											
Denver Developmental Screening Test	DDST	Cognition	NINFEA	x																	
Denver Developmental Screening Test	DDST	Cognition	RAINE	x	x	x															
Early Development Instrument	EDI	Cognition	MoBa						x												

Early Language Milestone Scale	ELM	Cognition	RAINE	x	x	x															
Gesell Developmental Observation-Revised	GDO-R	Cognition	RAINE	x	x																
Griffiths Mental Development scales	GMDS	Cognition	ALSPAC		x																
Griffiths Mental Development scales	GMDS	Cognition	RAINE	x	x																
Hawaii Early Learning Profile	HELP	Cognition	RAINE	x	x																
Hooper Visual Organization Test	HVOT	Cognition	CHOP									x									
Intelligence Quotient	IQ	Cognition	NFBC1966												x			x			
Matrix Reasoning Test	MRT	Cognition	NFBC1966																		x
McCarthy Scales of Children's Abilities	MSCA	Cognition	INMA					x													
McCarthy Scales of Children's Abilities	MSCA	Cognition	RHEA					x													
Mini Mental Status Examination	MMSE	Cognition	HBCS																		x
Question		Cognition	DNBC	x																	
Question		Cognition	DNBC		x																
Raven's Progressive Matrices	RPM	Cognition	BiB							x											
Raven's Progressive Matrices	RPM	Cognition	DNBC						x												
Raven's Progressive Matrices	RPM	Cognition	INMA								x		x	x							
Raven's Progressive Matrices	RPM	Cognition	RAINE										x								
Raven's Progressive Matrices	RPM	Cognition	RHEA							x											
Woodside Developmental Screening Chart		Cognition	RAINE	x	x																
Abstraction, Inhibition, and Working Memory Task	AIM	Executive functions	NFBC1966																		x
Ages & Stages Questionnaire	ASQ	Executive functions	EDEN				x	x													

Ages & Stages Questionnaire	ASQ	Executive functions	MoBa	x	x		x		x												
Ages & Stages Questionnaire	ASQ	Executive functions	RAINE	x	x		x														
Attention Network Test	ANT	Executive functions	BiB							x											
Attention Network Test	ANT	Executive functions	INMA								x		x	x							
Attention Network Test	ANT	Executive functions	MoBa									x									
Attention Network Test	ANT	Executive functions	RHEA							x											
Behavior Checklist (clinical rating)		executive functions	DNBC						x												
Behavior Rating Inventory of Executive Function	BRIEF	Executive functions	DNBC						x												
Behavior Rating Inventory of Executive Function	BRIEF	Executive functions	GECKO											x							
Behavior Rating Inventory of Executive Function	BRIEF	Executive functions	Generation R					x													
Cambridge Neuropsychological Test Automated Battery	CANTAB	Executive functions	SWS							x											
Children's Colour Trails Test	CCTT	Executive functions	CHOP									x									
Cogstate Brief Battery	CBB	Executive functions	HBCS																		x
Cogstate Brief Battery	CBB	Executive functions	HBCS																		x
Cogstate Brief Battery	CBB	Executive functions	RAINE													x					
Conners Kiddie Continuous Performance Test	KCPT	Executive functions	INMA					x													
Consortium to Establish a Registry for Alzheimer’s Disease Neuropsychological Battery	CERAD-NB	Executive functions	HBCS																		x
Continuous Performance Test	CPT	Executive functions	CHOP									x									
Counting Span Test	CST	Executive functions	ALSPAC											x							

Cups task		Executive functions	INMA											x							
Developmental Neuropsycholo gical Assessment, Second Editio	NEPSY-II	Executive functions	EDEN				x														
Developmental Neuropsycholo gical Assessment, Second Editio	NEPSY-II	Executive functions	Generation R								x										
Developmental Neuropsycholo gical Assessment, Second Editio	NEPSY-II	Executive functions	SWS					x													
Doctor diagnosis (self-reported)		Executive functions	NINFEA											x							
Habituation Task	NA	Executive functions	ALSPAC	x																	
Hungry Donkey Task	HDT	Executive functions	CHOP									x									
Kaufman Assessment Battery for Children	KABC II	Executive functions	CHOP									x									
McCarthy Scales of Children's Abilities	MSCA	Executive functions	INMA					x													
McCarthy Scales of Children's Abilities	MSCA	Executive functions	RHEA					x													
Montreal Cognitive Assessment	MoCa	Executive functions	HBCS																		x
Number Knowledge Test	NKT	Executive functions	EDEN						x												
Pair Cancellation Test		Executive functions	CHOP									x									
Reversal Digits Subtest		Executive functions	CHOP									x									
Stop Signal Task	SST	Executive functions	ALSPAC											x							
Stroop Interference Score		Executive functions	CHOP									x									
Symbol Digit Modalities Test	SDMT	Executive functions	CHOP									x									
Symbol Digit Modalities Test	SDMT	Executive functions	RAINE											x							

Teddy Bear Cancellation Test	TBCT	Executive functions	EDEN				x														
Test of Everyday Attention for Children	TEA-Ch	Executive functions	ALSPAC									x		x							
Test of Everyday Attention for Children	TEA-Ch	Executive functions	DNBC						x												
Trail Making Test	TMT	Executive functions	BiB							x											
Trail Making Test	TMT	Executive functions	HBCS																		x
Trail Making Test	TMT	Executive functions	INMA								x		x	x							
Trail Making Test	TMT	Executive functions	MoBa									x									
Trail Making Test	TMT	Executive functions	RHEA							x											
Visual Perceptual Skills (non-motor) test		Executive function	SWS					x													
Wechsler Preschool and Primary Scale of Intelligence	WPPSI	Executive functions	ALSPAC					x													
Wechsler Preschool and Primary Scale of Intelligence	WPPSI	Executive functions	DNBC						x												
Wechsler Preschool and Primary Scale of Intelligence	WPPSI	Executive functions	EDEN						x												
Wechsler Preschool and Primary Scale of Intelligence	WPPSI	Executive functions	SWS					x													
Working Memory Test	N-Back	Executive functions	ALSPAC														x				
Working Memory Test	N-Back	Executive functions	BiB							x											
Working Memory Test	N-Back	Executive functions	INMA								x		x	x							
Working Memory Test	N-Back	Executive functions	RHEA							x											
British Ability Scales	BAS	Intelligence	ELFE				x														
British Ability Scales	BAS	Intelligence	RAINE										x								
Cattell Infant Intelligence Scale	CIIS	Intelligence	RAINE	x	x																
Culture Fair Intelligence Test	CFIT	Intelligence	CHOP						x												

Non-Verbal Intelligence Test	SON-R	Intelligence	Generation R					x													
Parent Report of Children's Abilities	PARCA	Intelligence	Generation R			x															
Wechsler Abbreviated Scale of Intelligence	WAIS	Intelligence	ALSPAC													x					
Wechsler Abbreviated Scale of Intelligence	WAIS	Intelligence	DNBC						x												
Wechsler Abbreviated Scale of Intelligence	WAIS	Intelligence	SWS							x											
Wechsler Intelligence Scale for Children	WISC	Intelligence	ALSPAC									x									
Wechsler Intelligence Scale for Children	WISC	Intelligence	Generation R												x						
Wechsler Intelligence Scale for Children	WISC	Intelligence	INMA								x		x								
Wechsler Intelligence Scale for Children	WISC	Intelligence	RAINE										x								
Wechsler Memory Scale	WMS-III	Intelligence	NFBC1966																		x
Wechsler Preschool and Primary Scale of Intelligence	WPPSI	Intelligence	ALSPAC					x													
Wechsler Preschool and Primary Scale of Intelligence	WPPSI	Intelligence	DNBC						x												
Wechsler Preschool and Primary Scale of Intelligence	WPPSI	Intelligence	EDEN						x												
Wechsler Preschool and Primary Scale of Intelligence	WPPSI	Intelligence	SWS					x													
Auditory Analysis Test		Language	ALSPAC								x										
Australian Developmental Record for Infants and Young Children		Language	RAINE	x	x																
Battelle Developmental Inventory Screening Test	BDIST	Language	INMA					x													

Bayley Scales of Infant Development	BSID	Language	EDEN		x																
Bayley Scales of Infant Development	BSID	Language	INMA		x																
Bayley Scales of Infant Development	BSID	Language	RAINE	x	x																
Bayley Scales of Infant Development	BSID	Language	RHEA		x																
British Ability Scales	BAS	Language	ELFE				x														
British Ability Scales	BAS	Language	RAINE										x								
British Picture Vocabulary Scale	BPVS	Language	BiB					x													
Bus Story Test	BST	Language	ALSPAC						x												
Checklist of 20 Statements about Language-Related Difficulties	Språk20	Language	MoBa						x			x									
Children's auditory performance scale	CHAPS	Language	ALSPAC											x							
Children's Communication Checklist	CCC	Language	ALSPAC										x								
Children's Communication Checklist	CCC-2	Language	MoBa						x			x									
Children's developmental progress from birth to five years	STYCAR	Language	RAINE	x	x																
Children's Test of Nonword Repetition	CNRep	Language	ALSPAC									x									
Clinical Evaluation of Language Fundamentals	CELF	Language	RAINE										x								
Consortium to Establish a Registry for Alzheimer’s Disease Neuropsychological Battery	CERAD-NB	Language	HBCS																		x
Dale and Bishop language rating		Language	MoBa				x														
Denver Developmental Screening Test	DDST	Language	ALSPAC	x	x	x		x	x	x											

Denver Developmental Screening Test	DDST	Language	NINFEA	x																	
Denver Developmental Screening Test	DDST	Language	RAINE	x	x	x															
Developmental Neuropsychological Assessment, Second Editio	NEPSY-II	Language	EDEN				x														
Developmental Neuropsychological Assessment, Second Editio	NEPSY-II	Language	Generation R								x										
Developmental Neuropsychological Assessment, Second Editio	NEPSY-II	Language	SWS					x													
Doctor diagnosis (self-reported)		Language	NINFEA											x							
Early Language in Victoria Study	ELVS	Language	MoBa						x			x									
Early Language Milestone Scale	ELM	Language	RAINE	x	x	x															
Early Years Foundation Stage Profile	EYFSP	Language	BiB					x													
European oral language test battery of aphasic children	ELOLA	Language	EDEN				x														
FAS Animals Verbal Fluency Test	FAS	Language	CHOP									x									
Gesell Developmental Observation-Revised	GDO-R	Language	RAINE	x	x																
Griffiths Mental Development scales	GMDS	Language	ALSPAC		x																
Griffiths Mental Development scales	GMDS	Language	RAINE	x	x																
Hawaii Early Learning Profile	HELP	Language	RAINE	x	x																
Initial Consonants Detection Test		Language	ALSPAC						x												
Intelligibility/Complexity of 3-year-old Children’s Utterances		Language	MoBa				x														

Language skills		Language	NFBC1966	x	x	x	x	x	x												
Language skills		Language	NFBC1986								x										
Letter Identification		Language	BiB					x													
Loss of Skills		Language	MoBa				x														
MacArthur-Bates Communicative Development Inventories	MB-CDI	Language	ALSPAC	x	x		x			x											
MacArthur-Bates Communicative Development Inventories	MB-CDI	Language	EDEN			x															
MacArthur-Bates Communicative Development Inventories	MB-CDI	Language	ELFE		x	x															
MacArthur-Bates Communicative Development Inventories	MB-CDI	Language	Generation R		x																
McCarthy Scales of Children's Abilities	MSCA	Language	INMA					x													
McCarthy Scales of Children's Abilities	MSCA	Language	RHEA					x													
Montreal Cognitive Assessment	MoCa	Language	HBCS																		x
Multisyllabic Word Repetition		Language	ALSPAC						x												
Non-word repetition		Language	ALSPAC						x			x									
Object Naming Assessment		Language	ALSPAC			x															
Parent Report of Children's Abilities	PARCA	Language	Generation R			x															
Peabody Picture Vocabulary Test	PPVT	Language	RAINE						x				x								
Phonological production		Language	ALSPAC						x												
Phonological production		Language	BiB						x												
Question		Language	BiB					x													
Question		Language	DNBC		x				x		x			x							
Question		Language	MoBa		x		x		x		x	x									

Question		Language	MoBa						x												
Question		Language	MoBa						x												
Reynell Developmental Language Scale	RDLS	Language	ALSPAC		x	x			x												
Semantic Verbal Fluency Test	SVF	Language	INMA								x	x									
Speech and Language Assessment Scale	SLAS	Language	MoBa						x												
Taaltest Voor Kinderen	TVK	Language	Generation R						x												
Verbal Fluency Test	VFT	Language	NFBC1966																		x
Wechsler Objective Language Dimensions	WOLD	Language	ALSPAC									x									
Wechsler Objective Reading Dimensions	WORD	Language	ALSPAC								x										
Woodside Developmental Screening Chart		Language	RAINE	x	x																
California Verbal Learning Test	CVLT	Memory	NFBC1966																		x
Consortium to Establish a Registry for Alzheimer's Disease Neuropsychological Battery	CERAD-NB	Memory	HBCS																		x
Corsi		Memory	BIB								x	x	x								
Counting Span Test	CST	Memory	ALSPAC											x							
Developmental Neuropsychological Assessment, Second Editio	NEPSY-II	Memory	EDEN				x														
Developmental Neuropsychological Assessment, Second Editio	NEPSY-II	Memory	Generation R								x										
Developmental Neuropsychological Assessment, Second Editio	NEPSY-II	Memory	SWS					x													
Digit Span Test	DST	Memory	ALSPAC					x	x												
Digit Span Test	DST	Memory	NFBC1966																		x

Forward and backwards digit recall		Memory	BIB								x	x	x								
McCarthy Scales of Children's Abilities	MSCA	Memory	INMA					x													
McCarthy Scales of Children's Abilities	MSCA	Memory	RHEA					x													
Non-word repetition		Memory	ALSPAC						x			x									
Recall of Objects Test		Memory	CHOP									x									
Reversal Digits Subtest		Memory	CHOP									x									
Rey Auditory Verbal Learning Test	RAVLT	Memory	CHOP									x									
Visual Object Learning Test	VOLT	Memory	NFBC1966																		x
Wechsler Memory Scale	WMS-III	Memory	NFBC1966																		x
Working Memory Test	N-Back	Memory	ALSPAC														x				
Working Memory Test	N-Back	Memory	BiB							x											
Working Memory Test	N-Back	Memory	INMA								x		x	x							
Working Memory Test	N-Back	Memory	RHEA							x											
14 item measure		Mental health	ALSPAC	x																	
Adolescent Sexuality Activity Index	ASAI	Mental health	ALSPAC											x	x	x	x				
Alarm Distress Baby Scale	ADBB	Mental health	EDEN		x																
Arnett Inventory of Sensation Seeking	AISS	Mental health	ALSPAC											x	x		x	x			
Assessment of Adult – Child Interaction with the Etch-a-Sketch	AMCIES	Mental health	ALSPAC												x						
Autism Quotient Questionnaire	AQ	Mental health	RAINE															x			
Battelle Developmental Inventory Screening Test	BDIST	Mental health	INMA					x													
Beck Depression Inventory	BDI	Mental health	HBCS																		x
Beck Depression Inventory	BDI	Mental health	NFBC1966																		x

Beck Depression Inventory	BDI	Mental health	RAINE												x	x					
Behavior Rating Inventory of Executive Function	BRIEF	Mental health	DNBC						x												
Behavior Rating Inventory of Executive Function	BRIEF	Mental health	GECKO											x							
Behavior Rating Inventory of Executive Function	BRIEF	Mental health	Generation R					x													
Bipolar II scale	BSDS	Mental health	NFBC1966																		x
Body Image Scale	BIS	Mental health	RAINE												x						
Bradburn Happiness Scale (Affect Balance Scale)	ABS	Mental health	RAINE			x		x				x	x		x	x					
Brief Problem Monitor	BPM	Mental health	Generation R										x								
Bullying and Friendship Interview Schedule	BFIS	Mental health	ALSPAC									x		x	x						
California Preschool Social Competence Scale	CPSCS	Mental health	INMA					x													
Cambridge Hormones and Moods project Friendship questionnaire		Mental health	ALSPAC									x		x	x		x				
Cantril's ladder scale		Mental health	DNBC											x							
Cantril Self-Anchoring Striving Scale		Mental health	DNBC														x				
Carey Temperament Scales		Mental health	ALSPAC	x	x																
Center for Epidemiologic Studies Depression Scale	CES-D	Mental health	HBCS																		x
Child Behaviour Checklist	CBCL	Mental health	BiB							x											
Child Behaviour Checklist	CBCL	Mental health	CHOP									x									

Child Behaviour Checklist	CBCL	Mental health	Generation R		x		x		x				x								
Child Behaviour Checklist	CBCL	Mental health	INMA								x	x	x								
Child Behaviour Checklist	CBCL	Mental health	MoBa		x		x		x												
Child Behaviour Checklist	CBCL	Mental health	RAINE					x				x	x		x	x					
Child Behaviour Checklist	CBCL	Mental health	RHEA							x											
Childhood Autism Spectrum Test	CAST	Mental health	INMA					x													
Childhood Autism Spectrum Test	CAST	Mental health	MoBa						x												
Childhood Interview for DSM-IV Borderline Personality Disorder		Mental health	ALSPAC											x			x		x		
Clinical Interview Schedule - Revised	CISR	Mental health	ALSPAC														x				
Cognitive style questionnaire	CSQ	Mental health	ALSPAC														x				
Communication and Symbolic Behaviour Scales	CSBS	Mental health	MoBa		x																
Conflict Tactics Scale		Mental health	ALSPAC												x						
Denver Developmental Screening Test	DDST	Mental health	ALSPAC	x	x	x		x	x	x											
Denver Developmental Screening Test	DDST	Mental health	NINFEA	x																	
Denver Developmental Screening Test	DDST	Mental health	RAINE	x	x	x															
Depression Anxiety Stress Scales	DASS	Mental health	RAINE																x		
Development and Well-Being Assessment	DAWBA	Mental health	ALSPAC							x			x	x	x	x					
Development and Well-Being Assessment	DAWBA	Mental health	DNBC											x							
Diagnostic Interview Schedule for Children	DISC-IV/DSM	Mental health	INMA					x													

Diagnostic Interview Schedule for Children	DISC-IV/DSM	Mental health	MoBa				x														
Diagnostic Interview Schedule for Children	DISC-IV/DSM	Mental health	NINFEA					x													
Diagnostic Interview Schedule for Children	DISC-IV/DSM	Mental health	RAINE										x								
Doctor diagnosis (self-reported)		Mental health	NINFEA											x							
Dyadic Adjustment Scale	DAS	Mental health	RAINE										x		x	x					
Dysfunctional Attitude Scale	DAS	Mental health	ALSPAC														x				
Early Screening of Autistic Traits Questionnaire	ESAT	Mental health	MoBa		x		x														
Eating Disorder Examination	EDE	Mental health	RAINE												x						
Emotionality, Activity, and Sociability Temperament Survey	EAS	Mental health	ALSPAC				x	x	x												
Emotionality, Activity, and Sociability Temperament Survey	EAS	Mental health	MoBa		x		x		x												
Eriksen Flanker Task	EFT	Mental health	INMA											x							
General Anxiety Disorder - 7	GAD-7	Mental health	ALSPAC																x		
Generalized Self-Efficacy Scale	GSE	Mental health	HBCS																		x
Hardy-Gill resilience scale		Mental health	HBCS																		x
Harter's Self Perception Profile for Children		Mental health	ALSPAC									x									
Health records		Mental health	BIB	x	x	x	x	x	x	x	x	x	x	x							
Hypomania Checklist-32	HCL-32	Mental health	ALSPAC																x		
Hypomanic Personality Scale	HPS	Mental health	NFBC1966																		x
Infant Characteristics Questionnaire - 6 months form	ICQ-6	Mental health	MoBa	x																	

Infant-Toddler Social and Emotional Assessment	ITSEA	Mental health	MoBa				x														
International Personality Item Pool	IPIP	Mental health	ALSPAC												x						
Kessler Psychological Distress Scale	K10	Mental health	RAINE													x					
Life Orientation Test	LOT-R	Mental health	HBCS																		x
Life Orientation Test	LOT-R	Mental health	NFBC1966																		x
Loss of Skills		Mental health	MoBa				x														
Major Depression Inventory	MDI	Mental health	DNBC															x			
McKnight Risk Factor Survey	MRFS	Mental health	DNBC											x				x			
McMaster Family Assessment Device	FAD	Mental health	RAINE			x		x				x	x		x	x					
Measure created for ALSPAC		Mental health	ALSPAC												x						
Modified Checklist for Autism in Toddlers	M-CHAT	Mental health	ELFE		x																
Modified Checklist for Autism in Toddlers	M-CHAT	Mental health	MoBa		x		x														
National Registry data		Mental health	HBCS																		x
Neuroticism-Extroversion-Openness Personality Inventory	NEO-PI	Mental health	HBCS																		x
Non-Verbal Communication Checklist	NVCC	Mental health	MoBa		x		x														
Nowicki-Strickland Internal-External scale	NSIE	Mental health	ALSPAC									x					x				
Olweus Bullying Questionnaire		Mental health	ALSPAC												x						
Parent/Teacher Rating Scale for Disruptive Behaviour Disorders	RS-DBD	Mental health	MoBa									x									
Perceived Self-Efficacy Scale (Cowen's)	PSES	Mental health	RAINE			x									x	x					
Perceived Stress Scale	PSS	Mental health	RAINE													x					

Perceptual Aberration Scale	PAS	Mental health	NFBC1966																		x
PLIKS-Q		Mental health	DNBC															x			
Preschool Play Behaviour Scale	PPBS	Mental health	Moba						x												
Psychopathy Screening Device		Mental health	ALSPAC												x		x				
Psychosis-like symptoms measure	PLIKS	Mental health	ALSPAC											x	x		x		x		
Psychosis-like symptoms measure	PLIKS	Mental health	DNBC											x							
PTSD checklist 5	PCL-5	Mental health	ALSPAC																x		
Quality of Life Questionnaire	SF36	Mental health	HBCS																		x
Quality of Life Questionnaire	15-D	Mental health	NFBC1966																		x
Question		Mental health	MoBa		x		x		x		x	x									
Question		Mental health	MoBa		x		x														
Question		Mental health	MoBa		x		x		x												
Question		Mental health	MoBa				x		x			x									
Question		Mental health	MoBa				x				x										
Question		Mental health	MoBa				x		x		x	x									
Question		Mental health	MoBa						x		x	x									
Question		Mental health	MoBa								x										
Question		Mental health	MoBa								x										
Question		Mental health	MoBa						x			x									
Question		Mental health	DNBC															x			
Question		Mental health	DNBC															x			
Question		Mental health	DNBC															x			
Questionnaire of Eating and Weight Patterns-Parent Report	QEWP-P	Mental health	Moba						x			x									
Resilience scale		Mental health	HBCS																		x
Revised Conners' Parent Rating Scale	CPRS-R	Mental health	BiB							x											
Revised Conners' Parent Rating Scale	CPRS-R	Mental health	Generation R								x										

Revised Conners' Parent Rating Scale	CPRS-R	Mental health	INMA								x		x	x							
Revised Conners' Parent Rating Scale	CPRS-R	Mental health	MoBa						x												
Revised Conners' Parent Rating Scale	CPRS-R	Mental health	RHEA							x											
Revised Physical Anhedonia Scale	PhAS	Mental health	NFBC1966																		x
Revised Rutter Parent Scale for Preschool Children		Mental health	ALSPAC				x														
Revised Social Anhedonia Scale	SAS	Mental health	NFBC1966																		x
Rutter's Behaviour Scale	RB2	Mental health	NFBC1986								x										
Schizoidia Scale		Mental health	NFBC1966																		x
Screen for Child Anxiety Related Emotional Disorders	SCARED	Mental health	MoBa									x									
Self image profile for adolescents	SIP-A	Mental health	ALSPAC												x						
Self-perception Profile for Adolescents	SPPA	Mental health	RAINE												x						
Self-reported antisocial behavior for young children questionnaire		Mental health	ALSPAC									x									
Short Mood and Feelings Questionnaire	SMFQ	Mental health	ALSPAC										x	x	x	x	x	x	x		
Short Mood and Feelings Questionnaire	SMFQ	Mental health	MoBa									x									
Short Norwegian Hierarchical Personality Inventory for Children	NHiPIC-30	Mental health	MoBa									x									
Short OCD Screener	SOCS	Mental health	DNBC											x				x			
Social communication disorders checklist	SCDC	Mental health	ALSPAC							x			x	x	x	x					
Social Communication Questionnaire	SCQ	Mental health	MoBa		x		x					x									

Social Responsiveness Scale	SRS	Mental health	Generation R						x												
Spence Children Anxiety Scale	SCAS	Mental health	DNBC															x			
Stattin and Kerr Parental Monitoring Measure		Mental health	ALSPAC												x	x					
Strengths and Difficulties Questionnaire	SDQ	Mental health	ALSPAC					x					x	x	x	x					
Strengths and Difficulties Questionnaire	SDQ	Mental health	BiB					x													
Strengths and Difficulties Questionnaire	SDQ	Mental health	CHOP						x					x							
Strengths and Difficulties Questionnaire	SDQ	Mental health	DNBC						x		x			x				x			
Strengths and Difficulties Questionnaire	SDQ	Mental health	EDEN				x														
Strengths and Difficulties Questionnaire	SDQ	Mental health	ELFE				x		x												
Strengths and Difficulties Questionnaire	SDQ	Mental health	GECKO						x					x							
Strengths and Difficulties Questionnaire	SDQ	Mental health	INMA						x		x			x							
Strengths and Difficulties Questionnaire	SDQ	Mental health	MoBa				x					x									
Strengths and Difficulties Questionnaire	SDQ	Mental health	RAINE										x								
Strengths and Difficulties Questionnaire	SDQ	Mental health	RHEA					x													
Strengths and Difficulties Questionnaire	SDQ	Mental health	SWS				x														
Strengths and Weaknesses of ADHD symptoms and Normal behavior	SWAN	Mental health	NFBC1986													x					
Stress in Children	SiC	Mental health	DNBC											x							
Structured interview		Mental health	ALSPAC										x		x						
Subtypes of the Antisocial Behavior Questionnaire	STAB	Mental health	ALSPAC											x							

Symptom Check List -25	SCL-25	Mental health	NFBC1966																		x
Teacher Report Form	TRF	Mental health	Generation R							x											
Teacher Report Form	TRF	Mental health	RAINE										x								
Temperament and character inventory	TCI	Mental health	NFBC1966																		x
The Children's Eating Behaviour Questionnaire	CEBQ	Mental health	MoBa									x									
The Emotionality, Activity and Shyness Temperament Questionnaire	EAS	Mental health	MoBa		x		x														
The Trier Social Stress Test	TSST	Mental health	HBCS																		x
Toddler Temperament Questionnaire	TTQ	Mental health	RAINE	x																	
Toronto Alexithymia Scale	TAS	Mental health	NFBC1966													x					x
Tridimensional Personality Questionnaire	TPQ	Mental health	HBCS																		x
Tridimensional Personality Questionnaire	TPQ	Mental health	NFBC1966																		x
Volume of offending meas ure used in the Edinburgh Study of Youth Transitions and Crime		Mental health	ALSPAC												x			x	x		
Warwick-Edinburgh Mental Well-Being Scale	WEMWBS	Mental health	DNBC															x			
Ways of Coping checklist	WAYS	Mental health	NFBC1966																		x
Youth Risk Behavior Surveillance System	YRBSS	Mental health	ALSPAC												x	x	x	x			
Youth Risk Behavior Surveillance System	YRBSS	Mental health	DNBC															x			
Youth Self Report	YSR	Mental health	Generation R												x						
Youth Self Report	YSR	Mental health	NFBC1986													x					
Youth Self Report	YSR	Mental health	RAINE												x	x					

Ages & Stages Questionnaire	ASQ	Psychomotor	EDEN				x	x													
Ages & Stages Questionnaire	ASQ	Psychomotor	MoBa	x	x		x		x												
Ages & Stages Questionnaire	ASQ	Psychomotor	RAINE	x	x		x														
Australian Developmental Record for Infants and Young Children		Psychomotor	RAINE	x	x																
Backward balancing		Psychomotor	Generation R										x								
Bayley Scales of Infant Development	BSID	Psychomotor	EDEN		x																
Bayley Scales of Infant Development	BSID	Psychomotor	INMA		x																
Bayley Scales of Infant Development	BSID	Psychomotor	RAINE	x	x																
Bayley Scales of Infant Development	BSID	Psychomotor	RHEA		x																
Brunet-Lezine psychometric scale	BLS	Psychomotor	EDEN		x	x															
Children's developmental progress from birth to five years	STYCAR	Psychomotor	RAINE	x	x																
Clinical Kinematic Assessment Tool	CKAT	Psychomotor	BiB					x													
Denver Developmental Screening Test	DDST	Psychomotor	ALSPAC	x	x	x		x	x	x											
Denver Developmental Screening Test	DDST	Psychomotor	NINFEA	x																	
Denver Developmental Screening Test	DDST	Psychomotor	RAINE	x	x	x															
Developmental Coordination Disorder Questionnaire	DCDQ	Psychomotor	DNBC								x										
Developmental Coordination Disorder Questionnaire	DCDQ	Psychomotor	DNBC								x										
Finger Tapping Test	FTT	Psychomotor	BiB							x											
Finger Tapping Test	FTT	Psychomotor	Generation R										x								
Finger Tapping Test	FTT	Psychomotor	INMA								x		x	x							

Finger Tapping Test	FTT	Psychomotor	RHEA							x											
Gesell Developmental Observation-Revised	GDO-R	Psychomotor	RAINE	x	x																
Griffiths Mental Development scales	GMDS	Psychomotor	ALSPAC		x																
Griffiths Mental Development scales	GMDS	Psychomotor	RAINE	x	x																
Grooved Pegboard Test	GPT	Psychomotor	CHOP									x									
Grooved Pegboard Test	GPT	Psychomotor	NFBC1966																		x
McCarthy Scales of Children's Abilities	MSCA	Psychomotor	INMA					x													
McCarthy Scales of Children's Abilities	MSCA	Psychomotor	RHEA					x													
Motor skills		Psychomotor	ALSPAC												x						
Motor skills		Psychomotor	NFBC1966	x	x	x	x	x													
Motor skills		Psychomotor	NFBC1986		x						x					x					
Movement Assessment Battery for Children	M-ABC	Psychomotor	ALSPAC								x										
Movement Assessment Battery for Children	M-ABC	Psychomotor	DNBC						x												
Movement Assessment Battery for Children	M-ABC	Psychomotor	DNBC						x												
Peg Moving Task	PMT	Psychomotor	EDEN				x														
Purdue Pegboard Test	PPT	Psychomotor	RAINE											x							
Question		Psychomotor	DNBC						x												
Question		Psychomotor	DNBC	x	x				x												
Question		Psychomotor	MoBa	x								x									
Touwen Infant Neurological Examination	TouwenNE	Psychomotor	Generation R	x	x																
Woodside Developmental Screening Chart		Psychomotor	RAINE	x	x																
20 items		School performance	ALSPAC										x								
Child Behaviour Checklist	CBCL	School performance	BiB							x											

Child Behaviour Checklist	CBCL	School performance	CHOP									x									
Child Behaviour Checklist	CBCL	School performance	Generation R		x		x		x				x								
Child Behaviour Checklist	CBCL	School performance	INMA								x	x	x								
Child Behaviour Checklist	CBCL	School performance	MoBa		x		x		x												
Child Behaviour Checklist	CBCL	School performance	RAINE					x				x	x		x	x					
Child Behaviour Checklist	CBCL	School performance	RHEA							x											
CITO Scores		School performance	Generation R												x						
Early Development Instrument	EDI	School performance	MoBa									x									
Early Years Foundation Stage Profile	EYFSP	School performance	BiB					x													
Grades		School performance	NFBC1966													x	x				
Grades		School performance	NFBC1986														x				
Grades		School performance	NINFEA								x			x							
Key Stage 1 Assessment		School performance	BiB							x											
Maths test		School performance	ALSPAC									x	x		x						
National Pupil Database		School performance	ALSPAC						x		x			x		x	x				
Neale Analysis of Reading Ability	NARA II	School performance	ALSPAC										x								
Perceived school performance		School performance	DNBC											x							
Question		School performance	ALSPAC							x	x	x		x		x					
Question		School performance	ALSPAC								x	x		x							
Question		School performance	DNBC											x							
Question		School performance	EDEN									x									
Question		School performance	ELFE						x												
Question		School performance	MoBa						x												
Question		School performance	MoBa									x									
Question		School performance	NFBC1986								x					x					
Question		School performance	NFBC1986								x					x					

Question		School performance	NFBC1986								x					x					
Rutter's Behaviour Scale	RB2	School performance	NFBC1986								x										
Teacher Report Form	TRF	School performance	Generation R							x											
Teacher Report Form	TRF	School performance	RAINE										x								
Vineland Adaptive Behaviour Scale-II	VABS-II	School performance	MoBa									x									

eMaterials 1. Cohort study specific acknowledgements

ALSPAC

We are extremely grateful to all of the families who took part in ALSPAC, the midwives for their help in recruiting them, and the whole ALSPAC team, which includes interviewers, computer and laboratory technicians, clerical workers, research scientists, volunteers, managers, receptionists and nurses.

ALSPAC Data Dictionary

Please note that the study website contains details of all the data that is available through a fully searchable data dictionary and variable search tool:

http://www.bristol.ac.uk/alspac/researchers/our-data/

ALSPAC Ethical approval and informed consent

Ethical approval for the study was obtained from the ALSPAC Ethics and Law Committee and the Local Research Ethics Committees. Consent for biological samples has been collected in accordance with the Human Tissue Act (2004). Informed consent for the use of data collected via questionnaires and clinics was obtained from participants following the recommendations of the ALSPAC Ethics and Law Committee at the time.

For more information, kindly visit http://www.bristol.ac.uk/alspac/researchers/research-ethics/ or contact the Executive at alspac-exec@bristol.ac.uk

BIB

Born in Bradford is only possible because of the enthusiasm and commitment of the children and parents in Born in Bradford. We are grateful to all participants, health professionals and researchers who have made Born in Bradford happen.

CHOP

The authors would particularly like to thank all the cohort participants for their generous collaboration. Furthermore, thanks to all persons who designed and conducted the study, entered the data, and participated in the data analysis and who are represented by the European Childhood Obesity Trial Study Group participants: B Koletzko, V Grote, M Totzauer, K Gürlich, P Schwarzfischer, N Aumüller, V Luque, M Zaragoza-Jordana, N Ferré, J Escribano, R Closa-Monasterolo, A Xhonneux, JP Langhendries, E Verduci, E Riva, D Gruszfeld.

DNBC

The authors would like to thank the participants, the first Principal Investigator of DNBC Prof. Jørn Olsen, the scientific managerial team, and DNBC secretariat for being, establishing, developing and consolidating the Danish National Birth Cohort.

EDEN

The authors thank the cohort participants and the EDEN mother-child study group, whose members are: I. Annesi-Maesano, J.Y. Bernard, J. Botton, M.A. Charles, P. Dargent-Molina, B. de Lauzon-Guillain, P. Ducimetière, M. de Agostini, B. Foliguet, A. Forhan, X. Fritel, A. Germa, V. Goua, R. Hankard, B. Heude, M. Kaminski, B. Larroque†, N. Lelong, J. Lepeule, G. Magnin, L. Marchand, C. Nabet, F Pierre, R. Slama, M.J. Saurel-Cubizolles, M. Schweitzer, O. Thiebaugeorges.

ELFE

The authors are grateful to 1) the former members of the Elfe unit without whom the project would never have started: Henri Léridon, initiator and former Principal Investigator of the project, Stéphanie Vandentorren, Claudine Pirus, Corinne Bois and Ando Rakotonirina; 2) the expertise and assistance of members of the Elfe unit for support functions, 3) all the researchers who contribute to the projects as members of the Elfe thematic groups and especially their coordinators; 4) all the field

research assistants and interviewers; 5) and above all, all the Elfe families who have placed their confidence in us and given up their time to the study.

GECKO Drenthe

The authors are grateful to the families who took part in the GECKO Drenthe study, the midwives, gynaecologists, nurses and GPs for their help for recruitment and measurement of participants, and the whole team from the GECKO Drenthe study.

Generation R

The authors gratefully acknowledge the contribution of participants, research collaborators, general practitioners, hospitals, midwives, and pharmacies in Rotterdam.

HBCS

The authors would particularly like to thank all the cohort participants for their generous collaboration.

INMA

The authors would particularly like to thank all the participants for their generous collaboration. The authors are grateful to Silvia Fochs, Nuria Pey, Mireia Garcia, Maria Victoria Estraña, Maria Victoria Iturriaga, Cristina Capo and Josep LLuch for their assistance in contacting the families and administering the questionnaires.

MoBa

The authors are grateful to all the participating families in Norway who take part in this on-going cohort study.

NFBC1966 and 1986

The authors thank all cohort members and researchers who have participated in the NFBC studies. We also wish to acknowledge the work of the NFBC project centre.

NINFEA

The authors thank all families participating in the NINFEA cohort.

The RAINE Study

We would like to acknowledge the Raine Study participants and their families for their ongoing participation in the study and the Raine Study team for study co-ordination and data collection. We also thank the NHMRC for their long term contribution to funding the study over the last 30 years. The core management of the Raine Study is funded by The University of Western Australia, Curtin University, Telethon Kids Institute, Women and Infants Research Foundation, Edith Cowan University, Murdoch University, The University of Notre Dame Australia and the Raine Medical Research Foundation.

RHEA

The authors would particularly like to thank all the cohort participants for their generous collaboration.

SWS

The authors are grateful to the women of Southampton who gave their time to take part in the Southampton Women's Survey and to the research nurses and other staff who collected and processed the data.

eMaterials 2. Cohort study specific funding

ALSPAC

Core funding for the Avon Longitudinal Study of Parents and Children (ALSPAC) is provided by the UK Medical Research Council and Wellcome (217065/Z/19/Z) and the University of Bristol. A comprehensive list of grants funding is available on the ALSPAC website (http://www.bristol.ac.uk/alspac/external/documents/grant-acknowledgements.pdf). TC and AE work in a unit that is supported by the University of Bristol and UK Medical Research Council (MC_UU_00011/6). The funders had no role in the design of the study, the collection, analysis, or interpretation of the data; the writing of the manuscript, or the decision to submit the manuscript for publication. The views expressed in this paper are those of the authors and not necessarily those of any funder.

BIB

BiB receives core infrastructure funding from the Wellcome Trust (WT101597MA) and a joint grant from the UK Medical Research Council (MRC) and Economic and Social Science Research Council (ESRC) (MR/N024397/1). This study has received support from the British Heart Foundation (CS/16/4/32482), US National Institutes of Health (R01 DK10324), European Research Council under the European Union's Seventh Framework Programme (FP7/2007-2013) / ERC grant agreement no 669545, and National Institute for Health Research Applied Research Collaboration Yorkshire and Humber (NIHR200166). PMW receives funding from the National Institute for Health Research Applied Research Collaboration for Greater Manchester. The views expressed are those of the author(s), and not necessarily those of the NHS, the NIHR or the Department of Health and Social Care.

CHOP

The CHOP study has been carried out with partial financial support from the Commission of the European Community, specific RTD Programme "Quality of Life and Management of Living Resources", within the Fifth Framework Program (research grants no. QLRT-2001-00389 and QLK1-CT-200230582), the Sixth Framework Program (contract no. 007036), and Seventh Framework Programme (EarlyNutrition; grant agreement no. 289346), the EU H2020 project LIFECYCLE under grant no. 733206 and the European Research Council Advanced Grant META-GROWTH (ERC-2012-AdG – no.322605) and with financial support from Polish Ministry of Science and Higher Education (2571/7.PR/2012/2). This manuscript does not necessarily reflect the views of the Commission and in no way anticipates the future policy in this area. No funding bodies had any role in the study design, data collection and analysis.

DNBC

The Danish National Birth Cohort was established with a significant grant from the Danish National Research Foundation. Additional support was obtained from the Danish Regional Committees, the Pharmacy Foundation, the Egmont Foundation, the March of Dimes Birth Defects Foundation, the Health Foundation and other minor grants. The DNBC Biobank has been supported by the Novo Nordisk Foundation and the Lundbeck Foundation. Follow-up of mothers and children have been supported by the Danish Medical Research Council (SSVF 0646, 271-08-0839/06-066023, O602-01042B, 0602-02738B), the Lundbeck Foundation (195/04, R100-A9193), The Innovation Fund Denmark 0603-00294B (09-067124), the Nordea Foundation (02-2013-2014), Aarhus Ideas (AU R9-A959-13-S804), University of Copenhagen Strategic Grant (IFSV 2012), and the Danish Council for Independent Research (DFF – 4183-00594 and DFF - 4183-00152).

EDEN

The EDEN study was supported by Foundation for medical research (FRM), National Agency for

Research (ANR), National Institute for Research in Public health (IRESP: TGIR cohorte santé 2008 program), French Ministry of Health (DGS), French Ministry of Research, INSERM Bone and Joint Diseases National Research (PRO-A) and Human Nutrition National Research Programs, Paris-Sud University, Nestlé, French National Institute for Population Health Surveillance (InVS), French National Institute for Health Education (INPES), the European Union FP7 programmes (FP7/2007-2013, HELIX, ESCAPE, ENRIECO, Medall projects), Diabetes National Research Program (through a collaboration with the French Association of Diabetic Patients (AFD)), French Agency for Environmental Health Safety (now ANSES), Mutuelle Générale de l'Education Nationale a complementary health insurance (MGEN), French national agency for food security, French speaking association for the study of diabetes and metabolism (ALFEDIAM).

ELFE

The Elfe cohort received funding from the National Research Agency Investment for the Future program [ANR-11-EQPX-0038]; French National Institute for Research in Public Health (IRESP TGIR 2009-2001 program); Ministry of Higher Education and Research; Ministry of Environment; Ministry of Health; French Agency for Public Health; Ministry of Culture; and National Family Allowance Fund.

GECKO Drenthe

The GECKO Drenthe birth cohort was funded by an unrestricted grant of Hutchison Whampoa Ld, Hong Kong and supported by the University of Groningen, Well Baby Clinic Foundation Icare, Noordlease, Paediatric Association of The Netherlands and Youth Health Care Drenthe.

Generation R

The general design of the Generation R Study is made possible by financial support from the Erasmus MC, University Medical Centre, Rotterdam, Erasmus University Rotterdam, Netherlands Organization for Health Research and Development (ZonMw), Netherlands Organisation for

Scientific Research (NWO), Ministry of Health, Welfare and Sport and Ministry of Youth and Families. This project received funding from the European Union's Horizon 2020 research and innovation programme (LIFECYCLE, grant agreement No 733206, 2016; EUCAN-Connect grant agreement No 824989; ATHLETE, grant agreement No 874583). VJ received funding from a Consolidator Grant from the European Research Council (ERC-2014-CoG-648916). LD received funding from the European Union's Horizon 2020 co-funded programme ERA-Net on Biomarkers for Nutrition and Health (ERA HDHL) (ALPHABET project (no 696295; 2017), ZonMw The Netherlands (no 529051014; 2017)). HM received funding from Stichting Volksbond Rotterdam, the Dutch Brain Foundation (De Hersenstichting, project number GH2016.2.01) and the NARSAD Young Investigator Grant from the Brain & Behaviour Research Foundation grant number 27853 (Dr. El Marroun). The study sponsors had no role in the study design, data analysis, interpretation of data, or writing of this report.

HBCS

HBCS has been supported by grants from British Heart Foundation, Finska Läkaresällskapet, the Finnish Special Governmental Subsidy for Health Sciences, Academy of Finland, Samfundet Folkhälsan, Liv och Hälsa, Juho Vainio Foundation, Yrjö Jahnsson Foundation, The Diabetes Research Foundation, Finnish Foundation for Cardiovascular Research, the Signe and Ane Gyllenberg Foundation, EU FP7 (DORIAN) project number 278603, EU Horizon 2020 (DynaHealth) project number 633595 and EU Horizon 2020 (Lifecycle) project No 733206.

INMA

This study was funded by grants from the Instituto de Salud Carlos III (Red INMA G03/176) and the Generalitat de Catalunya-CIRIT (1999SGR 00241). INMA-Valencia was funded by Grants from UE (FP7-ENV-2011 cod 282957 and HEALTH.2010.2.4.5-1), Spain: ISCIII (G03/176; FIS-FEDER: PI09/02647, PI11/01007, PI11/02591, PI11/02038, PI13/1944, PI13/2032, PI14/00891, PI14/01687, and

PI16/1288; Miguel Servet-FEDER CP11/00178, CP15/00025, and CPII16/00051), and Generalitat Valenciana: FISABIO (UGP 15-230, UGP-15-244,and UGP-15-249). INMA-Gipuzkoa was funded by grants from the Instituto de Salud Carlos III (FISFIS PI06/0867, FISPS09/0009) 0867, Red INMA G03/176) and the Departamento de Salud del Gobierno Vasco (2005111093 and 2009111069) and the Provincial Government of Guipúzcoa (DFG06/004 and FG08/001). INM-Menorca was funded by grants from the Instituto de Salud Carlos III (Red INMA G03/176). This study was supported by funding from the European Community's Seventh Framework Programme (FP7/2007-206) under grant agreement no 308333—the HELIX project. JJ holds Miguel Servet-II contract (CPII19/00015) awarded by the Instituto de Salud Carlos III (Co-funded by European Social Fund "Investing in your future"). ML has received funding from the European Union's Horizon 2020 research and innovation programme under the Marie Skłodowska-Curie grant agreement No 707404. The opinions expressed in this document reflect only the author's view. The European Commission is not responsible for any use that may be made of the information it contains. MC holds a Miguel Servet fellowship (CP16/00128) funded by Instituto de Salud Carlos III and co-funded by European Social Fund "Investing in your future". CW received a Sara Borrell fellowship (CD18/00132) from the Instituto de Salud Carlos III. RG was supported by funding from the Instituto de Salud Carlos III (PI14/00891 and PI17/00663) and Alicia Koplowitz Foundation 2017. ML has held a Miguel Servet-II contract (MSII16/00051) awarded by the Instituto de Salud Carlos III (Co-funded by European Social Fund "Investing in your future"). SL This study was supported by grants from Instituto de Salud Carlos III (FIS-FEDER: 13/1944, 16/1288 and 19/1338; Miguel Servet-FEDER: CP15/0025). MG holds a Miguel Servet-II contract (CPII18/00018) awarded by the Instituto de Salud Carlos III (Co-funded by European Social Fund "Investing in your future").

MoBa

The Norwegian Mother, Father and Child Cohort Study is supported by the Norwegian Ministry of Health and Care Services and the Ministry of Education and Research. Jennifer R. Harris is

supported, in part, by The Research Council of Norway through its Centres of Excellence funding scheme, project nr. 262700.

NINFEA

The NINFEA cohort was initially funded by the Compagnia SanPaolo Foundation and the Piedmont Region. It received funding from European projects: CHICOS (FP7 grant number HEALTH-FP7-2009-241604, LifeCycle (H2020 grant number 733206), ATHLETE (H2020 grant number 874583).

NFBC1966 and NFBC1986

NFBC1966 received financial support from University of Oulu (grant numbers 65354 and 24000692), Oulu University Hospital (grant numbers 2/97, 8/97 and 24301140), Ministry of Health and Social Affairs (grant numbers 23/251/97, 160/97 and 190/97), National Institute for Health and Welfare, Helsinki (grant number 54121), Regional Institute of Occupational Health, Oulu (grant numbers 50621 and 54231) and ERDF European Regional Development Fund (grant number 539/2010 A31592). NFBC1986 received financial support from EU QLG1-CT-2000-01643 (EUROBLCS, grant number E51560), NorFA (grant numbers 731, 20056 and 30167) and USA / NIH 2000 G DF682 (grant number 50945). Financial support for data generation, research and supporting staff was received from the Academy of Finland (grants numbers: 104781, 120315, 129269, 1114194, 24300796, 285547 (EGEA)); University Hospital Oulu, Biocenter, University of Oulu, Finland (grant number: 75617); NIHM (grant number: MH063706, Smalley and Jarvelin for NFBC1986 data collection), Juselius Foundation; NFBC1966 genotyping by NHLBI (grant number: 5R01HL087679-02] through the STAMPEED program [grant number: 1RL1MH083268-01); NIH/NIMH (grant number: 5R01MH63706:02); the European Commission: EURO-BLCS, Framework 5 award QLG1-CT-2000-01643 (for NFBC1986 data collection), ENGAGE project and grant agreement HEALTH-F4-2007 (grant number: 201413); EU H2020-HCO-2004 iHEALTH Action (grant number: 643774), EU H2020-PHC-2014 DynaHealth Action (grant number: 633595); ALEC Action (grant number: 633212); ERDF

European Regional Development Fund (grant number: 539/2010 A31592); the Medical Research Council (MRC), UK (grant numbers: G0500539, G0600705, G1002319, MR/M013138/1), EU H2020-SC1-2016-2017 LifeCycle Action (grant number: 733206). The programme is currently funded by EU H2020-SC1-2016-2017 LifeCycle Action (grant number: 733206) and EU-H2020 EUCAN Connect (grant number: 824989).

The RAINE Study

The Raine Study has been funded by program and project grants from the Australian National Health and Medical Research Council, the Commonwealth Scientific and Industrial Research Organisation, Healthway and the Lions Eye Institute in Western Australia. The Raine Study Gen2-17 year follow-up was funded by the NHMRC Program Grant (Stanley et al, ID 353514) and the GWAS data from the Gen2-17 year follow-up was funded by the NHMRC (Huang et al, ID 1059711) grant. The Raine Study participation in LIFECYCLE was funded by a grant from the National Health and Medical Research Council, Australia (GNT114285). The University of Western Australia (UWA), Curtin University, the Raine Medical Research Foundation, the Telethon Kids Institute, the Women's and Infant's Research Foundation (KEMH), Murdoch University, The University of Notre Dame Australia and Edith Cowan University provide funding for the Core Management of the Raine Study. REF is a recipient of a National Health and Medical Research Council Early Career Fellowship.

RHEA

The "Rhea" project was financially supported by European projects (EU FP6-2003-Food-3-NewGeneris, EU FP6. STREP Hiwate, EU FP7 ENV.2007.1.2.2.2. Project No 211250 Escape, EU FP7-2008-ENV-1.2.1.4 Envirogenomarkers, EU FP7-HEALTH-2009- single stage CHICOS, EU FP7 ENV.2008.1.2.1.6. Proposal No 226285 ENRIECO, EU- FP7- HEALTH-2012 Proposal No 308333 HELIX) and the Greek Ministry of Health (Program of Prevention of obesity and neurodevelopmental

disorders in preschool children, in Heraklion district, Crete, Greece: 2011-2014; “Rhea Plus”: Primary Prevention Program of Environmental Risk Factors for Reproductive Health, and Child Health: 2012-15).

SWS

The SWS is supported by grants from the Medical Research Council, National Institute for Health Research Southampton Biomedical Research Centre, British Heart Foundation, University of Southampton and University Hospital Southampton National Health Service Foundation Trust, and the European Union’s Seventh Framework Programme (FP7/2007-2013), project EarlyNutrition (grant 289346). Study participants were drawn from a cohort study funded by the Medical Research Council and the Dunhill Medical Trust. HMI's salary is paid by the UK Medical Research Council. Mark Hanson is supported by the British Heart Foundation.
